# Emerging trends in long-acting sustained drug delivery for glaucoma management

**DOI:** 10.1007/s13346-024-01779-4

**Published:** 2025-01-09

**Authors:** Yin Ho So, Deepakkumar Mishra, Sandip Gite, Rahul Sonawane, David Waite, Rahamatullah Shaikh, Lalitkumar K. Vora, Raghu Raj Singh Thakur

**Affiliations:** 1 School of Pharmacy, Medical Biology Centre, Queen’s University Belfast, Belfast, UK; 2 Centre for Pharmaceutical Engineering Science, School of Pharmacy and Medical Sciences, University of Bradford, Bradford, BD7 1DP UK

**Keywords:** Ocular, Glaucoma, Sustained release, Long-acting, Drug delivery, Intraocular pressure

## Abstract

Glaucoma is an optic neuropathy in which progressive degeneration of retinal ganglion cells and the optic nerve leads to irreversible visual loss. Glaucoma is one of the leading causes of blindness. The pathogenesis of glaucoma is determined by different pathogenetic mechanisms, including increased intraocular pressure, mechanical stress, excitotoxicity, resistance to aqueous drainage and oxidative stress. Topical formulations are often used in glaucoma treatment, whereas surgical measures are used in acute glaucoma cases. For most patients, long-term glaucoma treatments are given. Poor patient compliance and low bioavailability are often associated with topical therapy, which suggests that sustained-release, long-acting drug delivery systems could be beneficial in managing glaucoma. This review summarizes the eye’s physiology, the pathogenesis of glaucoma, current treatments, including both pharmacological and nonpharmacological interventions, and recent advances in long-acting drug delivery systems for the treatment of glaucoma.

## Graphical Abstract

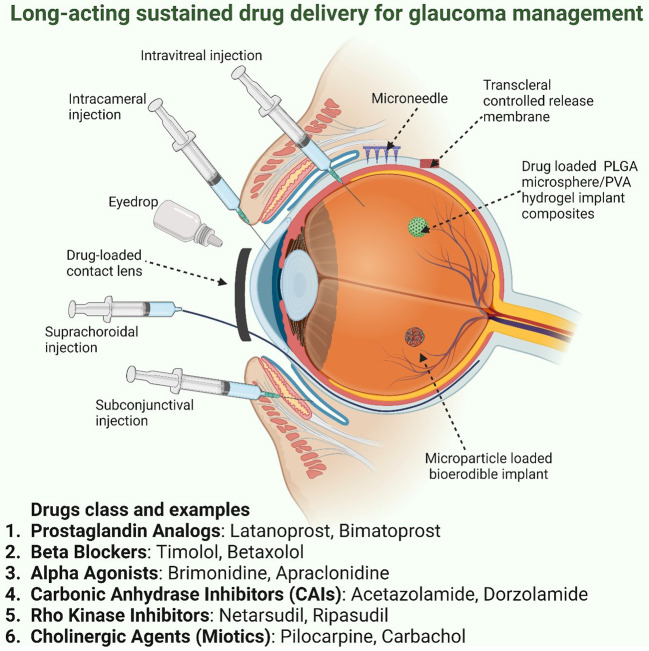

## Introduction

**Fig. 1:**
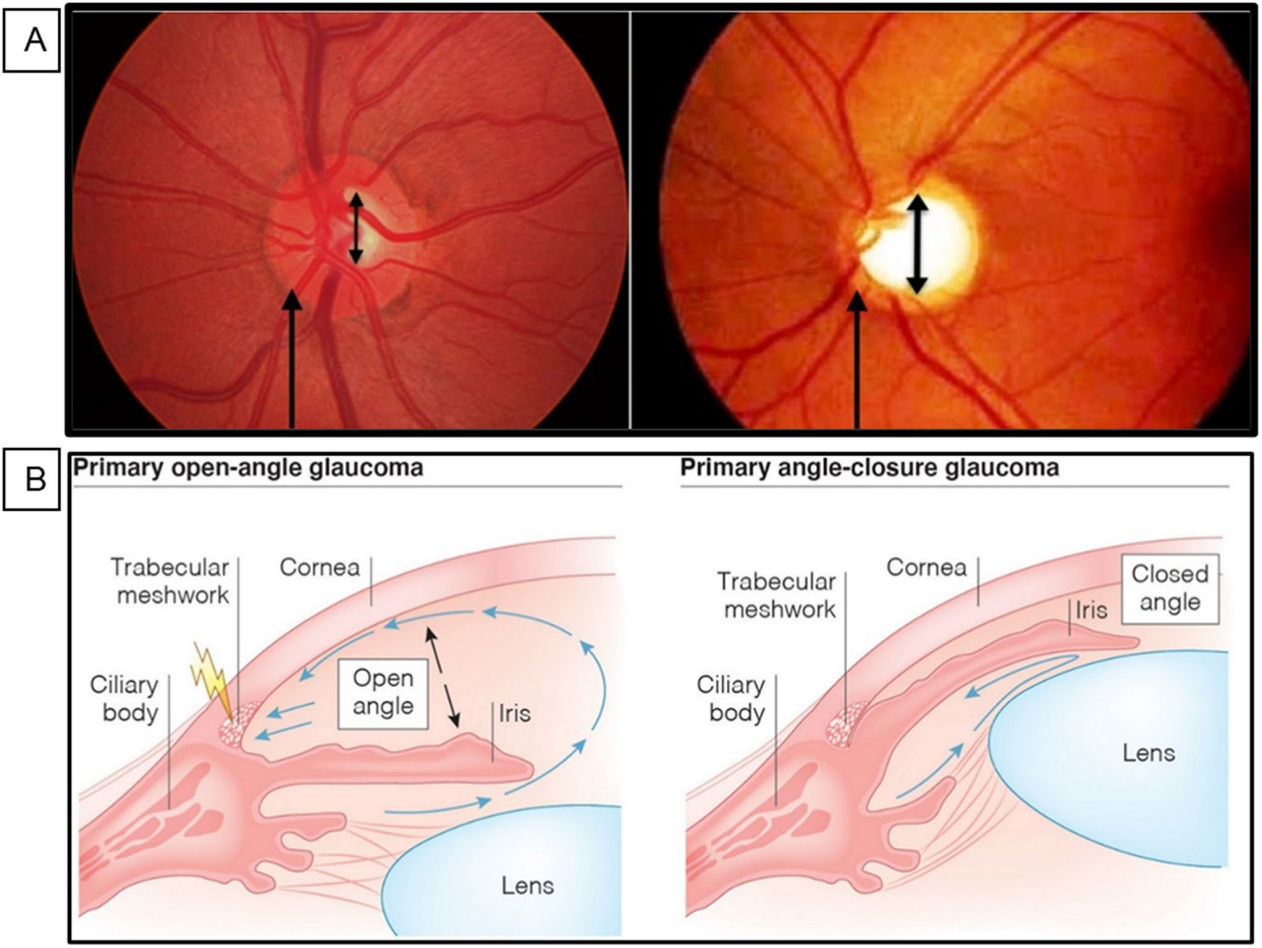
(**A**) Ophthalmoscopy image showing **(left)** a normal optic disc and **(right)** glaucomatous optic disc cupping. From: Adatia FA, Damji KF. Can Fam Physician. 2005;51(9):1229–1237. (**B**) The pathophysiology of **(left)** OAG and **(right)** ACG, taken from [8]

Glaucoma is an eye disease in which there is progressive damage to retinal ganglion cells (RGCs) that leads to vision loss [1]. Furthermore, the optic nerve and inner retinal neurons degenerate, leading to visual loss and the formation of optic disc cupping, as shown in Fig. 1A. Assessment for glaucoma is usually part of a routine sight test performed by an optometrist that allows early discovery of patients with glaucomatous optic nerve deformation and those who are prone to developing glaucoma. Intraocular pressure (IOP) is governed by ciliary body aqueous humor secretion and drainage, and an increase in IOP leads to the death of ganglion cells. The target of glaucoma management is focused on lowering IOP, which is the only changeable factor that can prevent further damage to the patient’s optic nerve [2]. There are two main types of glaucoma: open-angle glaucoma (OAG) and angle-closure glaucoma (ACG). The iridocorneal ‘angle’ describes the space between the anterior surface of the iris and the posterior surface of the cornea, where aqueous humor is naturally drained into the episcleral venous circulation via the trabecular meshwork. In the OAG, there is greater resistance to aqueous drainage through the trabecular meshwork, whereas in the ACG, drainage routes are blocked [3, 4]. Some posterior structures of the eye, such as the *lamina cribrosa* and adjacent tissues, are the weakest points where the optic nerve fibres exit [5]. Compression, deformation, and remodelling of the *lamina cribrosa* are due to mechanical stress and strain caused by the increase in IOP, leading to axonal damage and disturbance in axonal transport, which results in mitochondrial dysfunction of astrocytes and RGCs [6, 7]. Figure 1B shows the pathophysiology of OAG and ACG.

The global prevalence of glaucoma is 3.54% in people aged 40–80 years. Africans had the highest prevalence rate (4.20%) of OAG, and Asians had the highest prevalence rate (1.09%) of primary ACG. In 2020, approximately 76 million people (aged 40–80 years) were reported to suffer from glaucoma worldwide, which is predicted to increase to 111.8 million by 2040 [9, 10]. In another report that included eighty-one studies from 37 countries, with 216,214 participants, of which there were 5,266 OAG cases, black populations presented the highest rate (5.2% at 60 years) of OAG incidence, which increased to 12.2% at 80 years. The majority of OAG cases were reported among Hispanics (2.31%) and Caucasians (1.99%), with a prevalence of East and South Asians (1.48% and 1.56%, respectively). Men were 1.30% more likely to have OAG than women were. In 2015, 57.5 million people were affected by OAG globally, and 65.5 million were affected by OAG by 2020 [11]. In the UK, approximately 2% of people older than 40 years of age are estimated to have OAG, and this figure increases to nearly 10% in people older than 75 years of age. It is considered one of the most common causes of blindness in the UK, and statistics suggest that the number of people affected by this condition is expected to rise [12].

Treatment options differ depending on the type of glaucoma a patient presents with. For treating OAG, prostaglandin analogues, such as latanoprost, lower the IOP by increasing the uveoscleral outflow of the aqueous humor [13]. In contrast, beta-blockers, such as timolol maleate, are used to reduce aqueous humor production in the ciliary body [14]. Although ocular therapeutics can lower the IOP, surgical methods and laser treatment can often improve the condition to a greater degree. On the other hand, acute ACG is a medical emergency. In that scenario, those affected require medications to decrease the IOP as soon as possible. Laser treatment, namely, laser peripheral iridotomy, is most likely to proceed for those affected. In this process, a laser beam penetrates the iris and creates a minor hole to improve the drainage of aqueous humor in the anterior chamber. If the pressure remains high even after the iridotomy procedure, long-term medical treatment with topical agents such as β-blockers, α2–agonists, carbonic anhydrase inhibitors or prostaglandin analogues is necessary to control IOP [3]. Table 1 displays the treatment options for glaucoma, along with their respective mechanisms and potential adverse effects.

**Table 1:** Treatment options for glaucoma [15]

Class of drug	Examples	Mechanism of action	Local side effects
Prostaglandin analogues	Latanoprost, Travoprost, Bimatoprost, Tafluprost	First-line therapy, increase uveoscleral outflow	Change in eye color, change to eyelashes, eye redness
Beta-blockers	Timolol maleate, levobunolol hydrochloride, betaxolol	Reduce aqueous humor production	Eye discomfort, eye inflammation
Αlpha 2–agonists	Brimonidine tartrate, Apraclonidine*	Reduce aqueous humor production, increase uveoscleral outflow	Dry eye, eye disorders, asthenia, allergic reactions
Carbonic anhydrase inhibitors	Dorzolamide, Brinzolamide	Reduce aqueous humor production	Asthenia, eye discomfort, taste altered
Muscarinic-receptor agonists	Pilocarpine*	Increase aqueous outflow via trabecular meshwork	Bronchospasm, eye disorders, vitreous hemorrhage
Rho kinase inhibitors	Netarsudil**	Increase aqueous outflow	Eye discomfort, eye redness, temporary blurred vision
NO-donating prostaglandin analogues	Latanoprostene bunod**	Increase uveoscleral outflow and aqueous outflow via trabecular meshwork	Swollen eyelid, vision changes, eye irritation

* Unlicensed use in the UK

** Not launched in the UK

This review presents a comprehensive synthesis of recent advancements in long-acting drug delivery systems (LADDS) for glaucoma treatment, which have not been extensively covered in the literature. Specifically, we discuss emerging technologies such as nanoparticle-laden hydrogels, microtube-embedded contact lenses, and peptide-based hydrogels that provide significant improvements in sustained intraocular pressure reduction and increased bioavailability. Additionally, our review highlights recent clinical trials and preclinical studies on novel formulations, demonstrating their potential to revolutionize glaucoma management by extending drug release durations and minimizing patient compliance issues. This review, therefore, offers an updated perspective on the latest LADDS innovations and their clinical applications, distinguishing it from previously published works that have focused primarily on traditional approaches [16, 17]. It also provides a brief overview of the anatomy of the eye and the pathophysiology of glaucoma, followed by current treatment options, including both nonpharmacological and pharmacological measures.

## Eye anatomy, ocular barriers, and biomarkers

### The anatomy of the eye

**Fig. 2:**
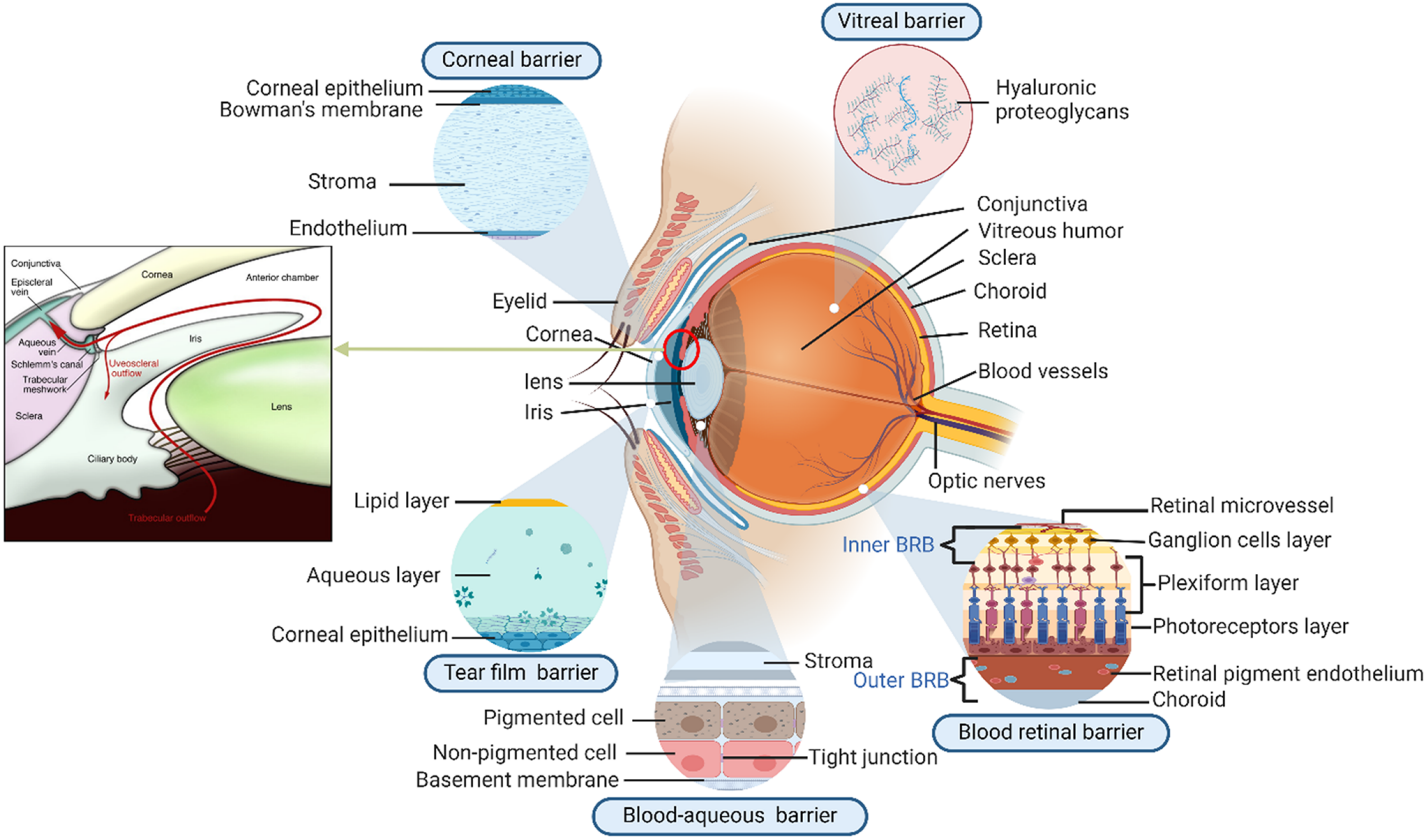
Physiological barriers in ocular drug delivery. Modified from Adrianto et al. [22]

The human eye is a nearly spherical-shaped organ. Its dimensions are reasonably constant, varying among healthy individuals by only a millimetre or two. The sagittal diameter is approximately 24 mm and is usually less than the transverse diameter. It weighs approximately 7.5 g and has a volume of approximately 6.5 cm^3^. The eye is composed of three distinguished layers, which enclose the anterior segment, lens, and vitreous body, as illustrated in Fig. 2. The outermost layer consists of the cornea, conjunctiva, aqueous humor, and sclera; the middle layer provides the primary source of blood to the eye and consists of the front to backwards iris, ciliary body, and choroid. The innermost region is composed of a vitreous body that provides optimal nourishment to the eye. This inner layer also consists of the optic nerve and the retina, which aligns with the choroid from the middle region [18]. The anterior chamber accounts for one-third of the eyeball volume, and it is the region of the eye between the lens and the cornea that consists of flowing aqueous humor [19]. Aqueous humor is the fluid produced in the eye that helps provide nourishment to the eye and surrounding tissues [20]. The light is refracted and transmitted to the lens by the cornea; internal and external stimuli are protected by the connective tissue coat called the sclera. The size of the pupil is controlled by the iris, whereas the ciliary body controls the shape and power of the lens. The retina is a processing center and a layered structure of neurons that captures and processes light [21].

### Ocular barriers

The eye consists of multiple barriers to prevent foreign matter from invading ocular tissues. From a drug delivery standpoint, these barriers pose significant challenges for drug entry into the eye and the subsequent treatment of ocular diseases. The blood‒aqueous barrier (BAB) is a significant obstacle in the eye that is associated with glaucoma. This barrier controls the transport of chemicals between blood vessels and aqueous humour, the fluid that occupies the anterior portion of the eye. Malfunction of the BAB can result in alterations in the makeup of aqueous humour. This can potentially contribute to an increase in IOP, which is a significant risk factor for the development of glaucoma.

Another significant obstacle is the blood‒retinal barrier (BRB), which controls the transportation of chemicals between blood vessels and the retina. Impairment of the BRB can result in changes in the flow of blood and metabolism in the retina, potentially causing the onset or advancement of glaucoma. The trabecular meshwork functions as an intraocular barrier that controls the drainage of aqueous humor from the eye. Dysfunction of the trabecular meshwork can impede the drainage of fluid, resulting in increased IOP and contributing to the onset or advancement of glaucoma.

Gaining insight into the function of these eye barriers in glaucoma will assist scientists in creating more effective therapies that specifically address these processes, thereby enhancing results for those affected by ailment [21].

#### Reflex blinking

On average, humans blink approximately 12 times per minute and tend to blink more rapidly when foreign matter irritates the eye [23]. A typical eye drop delivers 25–56 µL of a dosage form with a mean volume of 39 µL to the eye surface. However, an eye can hold briefly up to 30 µL, and the rest of the eye drop is eliminated via either nasolacrimal drainage or reflex blinking. These factors can therefore significantly reduce the overall amount of drug remaining on the ocular surface for treatment [24, 25].

#### Tear turnover

Following the administration of eye drops, the volume of cul-de-sac increases instantly, which causes rapid reflex blinking and increased secretion of tears, resulting in rapid drug loss from the ocular surface [25, 26]. The clearance rate of eye drops via the nasolacrimal duct is approximately 1 µL/min in humans [27]. Eventually, the elimination process is slowed until an average volume of 7–9 µL is reached [28]. Furthermore, tears are abundant in various types of proteins and electrolytes, which can impact the bioavailability of the drug. For example, some proteins, such as albumin and lysozyme, bind to drug molecules, which decreases the amount of free drug available for absorption. Additionally, electrolytes in tears maintain the pH and tonicity of the ocular surface. When an acidic or alkaline formulation is used, it can temporarily cause pH imbalance, which can irritate the eye. As a result, the blink rate and lacrimation are increased, and hence, the residence time of the drug decreases [29].

#### Nasolacrimal drainage

Nasolacrimal drainage is another barrier for topically applied formulations. Approximately 95% of an instilled formulation is eliminated via the nasolacrimal duct and conjunctiva [30]. This lacrimal drainage route leads to the nasal cavity, and histologically, the nasolacrimal duct and lacrimal sac are highly vascularized. Some portion of the instilled formulation is absorbed by this route and enters the systemic circulation, which may induce unwanted side effects [31].

#### Corneal epithelium

The corneal epithelium consists of a basal layer of columnar cells, two to three layers of wing cells and one or two outer layers of squamous cells [32]. This epithelium covers the front of the cornea and serves as a barrier to drug absorption from the topical formulation. Intercellular tight junctions enclose the layers of superficial cells, which restricts the penetration of drug molecules through the paracellular route [33]. The topical route is limited to hydrophilic drugs because of the presence of tight junctions between adjacent superficial epithelial cells. The corneal route allows small lipophilic drugs into the aqueous and anterior uvea, whereas the noncorneal or conjunctival/scleral route allows delivery of hydrophilic and larger molecules. Owing to aqueous turnover, the passage of drugs from the anterior to the posterior segments of the eye, such as the retina, vitreous and choroid, is not very efficient. Therefore, the posterior segments do not receive drugs through the ocular surface, and the drugs usually do not reach the back of the eye to provide sufficient concentrations to be therapeutic [34, 35].

### Biomarkers

In addition to IOP, other risk factors, such as age, sex, ethnicity, levels of oxidative stress, systemic and vascular factors, glutamate and nitric oxide levels and autoimmune disorders, are also involved in the onset of glaucoma [36]. Reports suggest that the increase in stress-related proteins such as crystallins and heat shock proteins can be used as proteomic biomarkers for primary OAG [37, 38]. Other stress-related proteins, such as ADP/ATP translocase 3, methyl-CpG-binding protein 2, PC4 and SRFS1-interacting protein 1, are closely associated with the development of glaucoma [39]. The changes in the expression level of immunoglobulin G in the aqueous humor also suggest the involvement of autoimmunity in the propagation of the primary OAG. The further downregulation of antibodies against αB-crystallin or vimentin, along with the upregulation of autoantibodies against proteins such as alpha-fodrin, HSP70 or myelin basic protein, suggests a greater role of autoimmunity in the progression of disease [36].

To enhance the practical application of these biomarkers in clinical settings, it is essential to consider how they can inform diagnosis and treatment strategies. For example, the identification of specific proteomic biomarkers could facilitate early detection of glaucoma, allowing timely intervention [40]. Clinicians could utilize these biomarkers in conjunction with traditional diagnostic methods, such as visual field tests and imaging studies, to improve diagnostic accuracy. Moreover, understanding the role of oxidative stress and autoimmune responses may lead to targeted therapies that address these underlying mechanisms [41].

Biomarkers such as crystallins and heat shock proteins not only serve as indicators of disease presence but also may reflect the severity of glaucomatous damage, providing a basis for monitoring disease progression and treatment efficacy. By integrating biomarker analysis into routine clinical practice, healthcare providers could personalize treatment plans on the basis of individual biomarker profiles, potentially increasing patient outcomes and reducing the risk of vision loss associated with glaucoma. Thus, expanding the discussion on biomarkers to include their practical implications in diagnosis and treatment is crucial for advancing glaucoma management [42].

## Current treatments for glaucoma

Glaucoma is a leading cause of irreversible vision loss characterized by the progressive degeneration of RGCs and the optic nerve, with increased IOP being a significant risk factor. The pathogenesis involves various mechanisms, including oxidative stress, excitotoxicity, and mechanical stress, which contribute to RGC death. Current treatment strategies focus primarily on lowering IOP through pharmacological and surgical interventions. However, traditional topical therapies often face challenges such as poor patient compliance and low bioavailability, highlighting the need for innovative approaches. Long-acting drug delivery systems, which provide sustained release of therapeutic agents, present promising solutions to enhance treatment efficacy and patient adherence. These systems can include ocular implants, nanoparticles, and hydrogels, which not only improve drug availability but also have the potential to integrate neuroprotective strategies aimed at preserving RGC function. Emerging therapies, such as neurotrophic factors and N-methyl-D-aspartate (NMDA) receptor antagonists, are being explored to mitigate RGC damage and promote survival. As research advances, the combination of long-acting drug delivery systems with neuroprotective agents may offer a comprehensive approach to managing glaucoma, addressing both IOP control and the preservation of visual function.

### Nonpharmacological treatments

Surgical interventions are sometimes required to treat glaucoma because some patients cannot reach the target IOP with maximum tolerated doses. An increasing number of patients are becoming intolerant to topical eye drops because of the high doses of drugs and preservatives used. All surgical procedures for glaucoma are intended to reduce IOP. This can be achieved in two ways: the reduction of aqueous inflow, namely, cyclodestruction, or the enhancement of aqueous outflow by external or internal filtration surgery. According to practice guidelines by the European Glaucoma Society [43, 44], external or internal filtration surgery is the first choice when drugs and laser therapy are unable to optimize the target IOP in patients with advanced-stage glaucoma. The most popular technique for cyclodestruction is transscleral diode laser cyclophotocoagulation (TCP). Alternatively, endoscopic diode laser cyclophotocoagulation (ECP) has higher success rates. MicroPulse lasers are used as a source for TCP [45, 46]. The first option for treating OAG is selective laser trabeculoplasty since it has similar hypotensive power to that of topical prostaglandins. Other laser techniques include micropulse diode laser trabeculoplasty, argon laser trabeculoplasty and titanium sapphire laser trabeculoplasty [47].

External and internal filtration are the conventional techniques that enhance aqueous outflow, particularly with microinvasive procedures. Trabeculectomy (TRAB) is the preferred antiglaucomatous surgical procedure among conventional external filtering operations and is considered the gold standard [48]. This technique involves creating a drainage hole in the sclera to allow the aqueous humor to exit the eye, effectively reducing IOP and preventing further optic nerve damage. New wound-healing modulators for trabeculectomy include antivascular endothelial growth factor agents and collagen matrix implants, such as OloGen [49]. Despite its effectiveness, TRAB carries risks, including complications such as hypotony and bleb leaks, which can be mitigated through the use of antifibrotic agents during surgery. Deep sclerectomy is a technique for nonpenetrating glaucoma surgery and is a good option in OAG eyes with previous failed filtering surgeries [50]. As the field of glaucoma surgery evolves, TRAB remains a vital option, particularly for patients with advanced or rapidly progressing disease, who require significant IOP reduction to preserve vision.

Nonpenetrating TRAB procedures include viscocanalostomy (VC) and canaloplasty; both techniques are effective for OAG [51]. However, both have lower initial success rates since they require a learning curve [52]. VC with phacoemulsification was reported to be an effective mitomycin C TRAB at 1 year [53, 54]. Additionally, the latest devices, such as iStent, CyPass and Trabectome, are used to reduce IOP. iStent involves a small titanium implant that is placed in Schlemm’s canal through the angle region, thus allowing bypass of increased trabecular resistance [55]. The CyPass microstent device is a small tube with tiny holes designed to drain fluid that is surgically placed in the eye in the suprachoroidal space [56]. The trabectome is the device that removes and aspirates the trabeculae and the inner wall of Schlemm’s canal [57]. Finally, excimer laser trabeculotomy reduces IOP by making small perforations in the trabecular meshwork and the inner wall of Schlemm’s canal [58]. Table 2 summarizes the nonpharmacological treatments mentioned above.

**Table 2:** Nonpharmacological treatments for glaucoma [59]

Treatment	Advantages	Disadvantages
Transscleral diode laser cyclophotocoagulation (TCP)	Effective for cyclodestruction	Invasive procedure
Endoscopic diode laser cyclophotocoagulation (ECP)	Higher success rates than TCP	More invasive than TCP
Selective laser trabeculoplasty	Similar hypotensive power to topical prostaglandins	May need to be repeated
Trabeculectomy (TRAB)	Gold standard for external filtering operations	Risk of complications, requires skilled surgeon
Deep sclerectomy	Good option for OAG eyes with previous failed filtering surgeries	Lower initial success rates, learning curve required
Viscocanalostomy (VC) and canaloplasty	Effective for OAG	Lower initial success rates, learning curve required
iStent	Minimally invasive, bypasses trabecular resistance	Limited long-term data
CyPass microstent	Drains fluid through suprachoroidal space	Limited long-term data
Trabectome	Removes and aspirates trabeculae and inner wall of Schlemm’s canal	Invasive procedure
Excimer laser trabeculotomy	Makes small perforations in trabecular meshwork	Limited long-term data

### Pharmacological treatment

The pharmacological treatments for glaucoma include five classes of topical drugs: prostaglandin analogues (PGAs), beta-adrenergic blockers (BBs), alpha-adrenergic agonists (AAs), carbonic anhydrase inhibitors (CAIs) and cholinergic agonists [60]. PGAs, such as latanoprost and travoprost, decrease IOP by improving uveoscleral aqueous outflow; PGAs bind to prostaglandin FP receptors in the ciliary muscle, resulting in relaxation of the muscle and increased uveoscleral outflow of aqueous humor [61]. They are thought to stimulate the synthesis of matrix metalloproteinases that dissolve the extracellular matrix of the ciliary muscle, thus reducing IOP [62]. PGAs have relatively poor corneal penetration, and their efficacy may be reduced in patients with thick corneas [63].

In contrast, BBs such as timolol maleate lower IOP by reducing aqueous humor production through the inhibition of beta-adrenergic receptors in the ciliary epithelium. They do not affect the outflow pathways or pupil size or accommodation [64]. By comparing the two classes, PGAs provide better 24-hour IOP control with a flatter diurnal curve compared to BBs and other IOP-lowering medications [65]. Additionally, PGAs have the advantages of once-daily dosing and a favourable adverse effect profile [61]. However, some patients may not respond adequately to PGA treatment because genetic factors affect drug metabolism and receptor sensitivity [62–64].

AAs and CAIs are the second-line choices of therapy. AAs reduce aqueous production and improve uveoscleral aqueous outflow. Some newer AAs, such as apraclonidine and brimonidine, target α2-adrenoreceptors more specifically, which causes fewer systemic hypotensive side effects than previous nonselective AAs. They act on α2-adrenergic receptors in the ciliary epithelium, inhibiting adenylyl cyclase and decreasing cAMP levels. This leads to reduced active transport of sodium and chloride ions into the posterior chamber, thereby decreasing aqueous humor formation. The activation of α2-receptors may also stimulate matrix metalloproteinases in the ciliary muscle, which breakdown extracellular matrix components. This remodelling of the ciliary muscle increases uveoscleral outflow and lowers IOP. By reducing aqueous humor production and enhancing uveoscleral outflow, AAs effectively lower IOP and aim to slow the progression of glaucomatous vision loss [66, 67]. Apraclonidine is ideal for short-term adjunctive therapy, whereas brimonidine is used for long-term treatment. The frequent use of topical AAs can cause local allergic reactions [68]. CAIs, such as dorzolamide and brinzolamide, also lower IOP but through different mechanisms. They inhibit the carbonic anhydrase enzyme in the ciliary epithelium, which catalyzes the reversible hydration of carbon dioxide. This reduces the production of bicarbonate ions, decreasing the active transport of sodium, bicarbonate and fluid into the posterior chamber, thereby reducing aqueous humor formation [69, 70]. Combination therapy can be beneficial in glaucoma treatment. Common combinations such as PGA-BB, AA-BB or CAI-BB are used to improve patient compliance, avert washout effects, simplify dosages, and limit exposure to formulation preservatives [71].

Cholinergic agonists such as pilocarpine and echothiophate iodide lower IOP by increasing conventional trabecular outflow and are used as adjunctive therapies in the treatment of OAG. Pilocarpine is a direct-acting cholinergic agonist that binds directly to muscarinic receptors. Echothiophate iodide, on the other hand, is an indirect-acting cholinergic agent that works by inhibiting acetylcholinesterase, thereby increasing the concentration of acetylcholine at receptor sites and prolonging its effects. These medications are used as adjunctive therapies in OAG when first-line treatments such as prostaglandin analogues or beta-blockers are insufficient to control IOP [72]. They are also indicated to prevent angle closure in cases of angle narrowing after neodymium-aluminum-garnet iridotomy or laser iridoplasty procedures. Although IOP is effectively reduced in OAG eyes, cholinergic agonists can cause significant side effects, including miosis, blowache, induced myopia and retinal detachment [73], and their use has declined in recent years because of the development of more effective and better-tolerated medications with fewer side effects.

Oral CAIs are sometimes added to the treatment protocol if the IOP is acutely elevated or untreated with topical agents, as is the case in acute ACG. They are rapidly reversible and are the best replacement for topical drugs in cases of intolerance, particularly if any possible future surgery has been planned. Oral CAIs can have undesired side effects, such as loss of appetite, fatigue, peripheral paresthesia, and nephrolithiasis [74].

Nitric oxide (NO)-donating moieties have recently become attractive candidates for some current APIs to enhance therapy. The key features of the NO-donating moiety are its anti-inflammatory properties, antiplatelet properties, and improved vasodilation [75]. For ocular use, the NO-donating moiety has the potential to reduce IOP by relaxing the trabecular meshwork; however, there is a limited understanding of either the toxicity of NO to ocular tissues or the amount of NO that would be required to relax the trabecular meshwork effectively. Additionally, owing to the nature of NO, it offers only a short ocular resistance time and poor penetration through the cornea; therefore, it could be used as an additive to antiglaucoma drugs to enhance glaucoma therapy. For example, NCX 434, an NO-donating triamcinolone acetate compound, was developed to improve retinal vasculature vasodilation and oxygenating optic nerve heads in a glaucomatous cynomolgus monkey model [76].

Latanoprost is typically a first-line IOP-reducing agent for the treatment of OAG by increasing uveoscleral outflow. Although effective, patients often require adjunctive therapies to maintain their IOP targets and delay the progression of glaucoma. The latanoprostene bunod (Vyzulta™), which was developed by Bausch and Lomb and the FDA in 2017, integrates a novel NO-donating moiety with a prominent antiglaucoma agent. A study revealed that the efficacy of latanoprostene was dose dependent and that the marketed concentration of 0.024% v/v was the minimal dose to achieve the greatest reduction in diurnal IOP compared with conventional 0.005% v/v latanoprost eye drops. Furthermore, in vivo studies of latanoprostene bunods have shown limited safety issues, i.e., neither blurred vision nor pupil dilation have been reported as side effects. In addition, the NO component of the latanoprostene bundle targets Schlemm’s canal and the trabecular meshwork directly to achieve an additional IOP drop compared with conventional latanoprost eye drops [77].

Neuroprotection is a critical aspect of glaucoma treatment that warrants further exploration. It is an alternative therapy aimed at protecting the optic nerve and preventing the death of RGCs, which are characterized by progressive degeneration leading to irreversible vision loss [78, 79]. The mechanisms underlying RGC death include oxidative stress, mitochondrial dysfunction, excitotoxicity, and neuroinflammation. Current neuroprotective strategies aim to mitigate these processes and preserve RGC function by modifying cellular factors related to the optic nerve or eliminating risk factors that damage it.

Both synthetic compounds and natural supplements can provide neuroprotection in various ways [80, 81]. Among the existing approaches, antioxidants such as vitamin E, coenzyme Q10, omega-3 fatty acids, and ginkgo biloba extract have shown promise in reducing oxidative damage and improving ocular blood flow [82, 83]. Additionally, NMDA receptor antagonists, including memantine, are being investigated for their potential to protect RGCs from excitotoxic injury by blocking NMDA receptors [84, 85], [81, 82]. Neurotrophic factors, such as brain-derived neurotrophic factor and ciliary neurotrophic factor, offer another avenue for neuroprotection by promoting RGC survival and function. Delivering these neurotrophins to the retina may compensate for target-derived neurotrophin deprivation [86] Emerging therapies, including stem cell treatments and gene therapies targeting neuroprotective pathways, hold promise for future applications in glaucoma management.

An important advantage of the neuroprotective strategy is that it aims to preserve both the functional and structural characteristics of RGCs to maintain useful vision. Successful neuroprotection requires slowing or preventing RGC death while maintaining the electrical, biochemical, and structural integrity needed for RGC function and relationship with surrounding cells [86, 87]. However, the development of effective neuroprotective therapies faces challenges, including the need for targeted delivery and integration with existing IOP-lowering treatments. Additionally, few randomized, double-masked, controlled clinical trials have demonstrated significant beneficial effects of neuroprotective agents compared with no intervention in patients with glaucoma. Ongoing research is focused on optimizing neuroprotective strategies and demonstrating their efficacy in preserving vision in glaucoma patients [88].

## Long-acting drug delivery systems for the treatment of glaucoma

The aim of antiglaucoma therapy is to lower IOP to 18 mmHg at all clinical visits or to maintain an average IOP of 12.3 mmHg, leading to a halt in glaucoma progression, followed by anti-inflammatory and neuroprotective treatments. This would typically require follow-up appointments every 6–12 months depending on the patient’s condition and associated risk factors [89]. One of the major drawbacks of the currently available treatment regimens and the topical route of administration is the need for frequent clinic visits, which can lead to a loss of patient compliance. Up to 80% of patients fail to instill eye drops on a regular basis, leading to worsening of the disease [90]. Overall, patient noncompliance with dosage regimens leads to worsening of disease and an increase in the already enormous burden on healthcare systems.

**Fig. 3:**
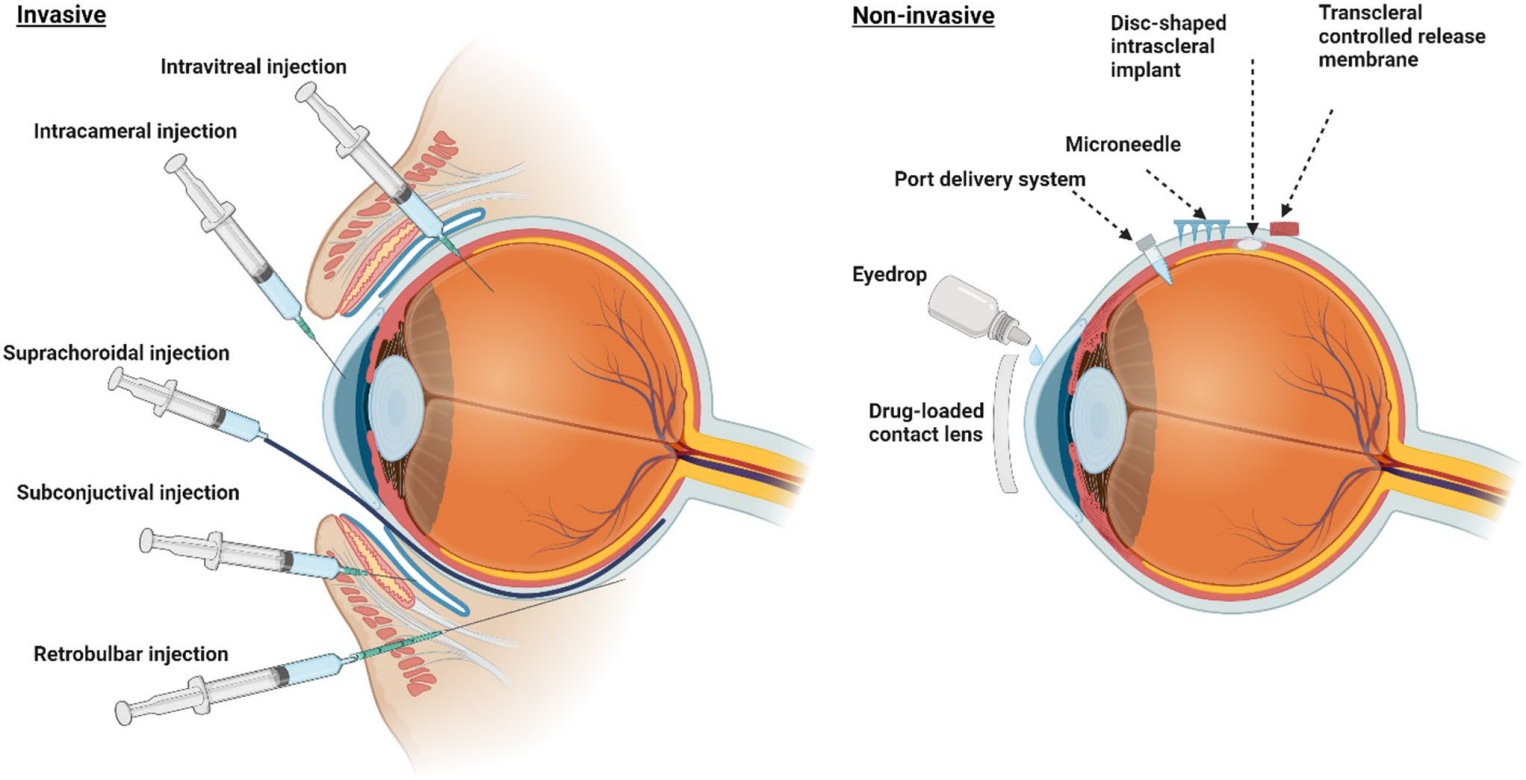
Different routes for the administration of long-acting drug delivery systems: **(Left)** invasive route **(Right)** noninvasive route

To overcome the challenges of current therapies, several researchers are focusing on developing innovative, minimally invasive, patient-compliant and low-cost long-acting drug delivery systems (LADDSs). LADDSs could provide a sustained and prolonged therapeutic concentration of drug molecules, thus improving the therapeutic response, limiting visits to the clinic, and improving patient compliance and adherence. LADDSs can be delivered to the eye via topical, intracameral, intravitreal or periocular routes (Fig. 3) in the form of nano/microparticles, hydrogels, inserts, drug-loaded contact lenses, or preformed implants for effective management of glaucoma.

The design and performance of an LADDS are highly dependent on the physicochemical properties of the therapeutic drug, the target disease, the duration of drug delivery and the site of administration/implantation. Some of the promising LADDSs in the literature for the treatment of glaucoma are discussed in the following sections.

### Topical LADDS

The topical route of drug administration is the most accessible and preferred route for patients. Historically, eye drops have been used for the management of anterior chamber disease for more than 100 years [91]. Topical eye drops have been associated with limited bioavailability (< 5%), which is often translated into shorter residence times and fluctuations in therapeutic concentrations. Several topical LADDSs, such as drug-loaded contact lenses, punctum plugs, hydrogels, and particulate-based systems, have been developed to allow the sustained release of drugs to offer better control over pharmacokinetic profiles.

#### Contact lenses

Contact lenses are ocular prosthetics regulated by the US Food and Drug Administration (FDA) and are mostly used for the correction of refractory errors. Although first manufactured by Thomas Young in 1801, contact lenses gained popularity in the 1960s after the advent of polymethyl methacrylate (PMMA)-based contact lenses [92]. Depending on the material used in fabrication, there are two major types of contact lenses: rigid gas-permeable contact lenses and soft contact lenses that are made of either hydrogel or silicon hydrogel [93]. Soft contact lenses account for 87% of clinical use, as they are preferable over rigid gas-permeable contact lenses, mainly because of the properties of the materials used [94].

**Fig. 4:**
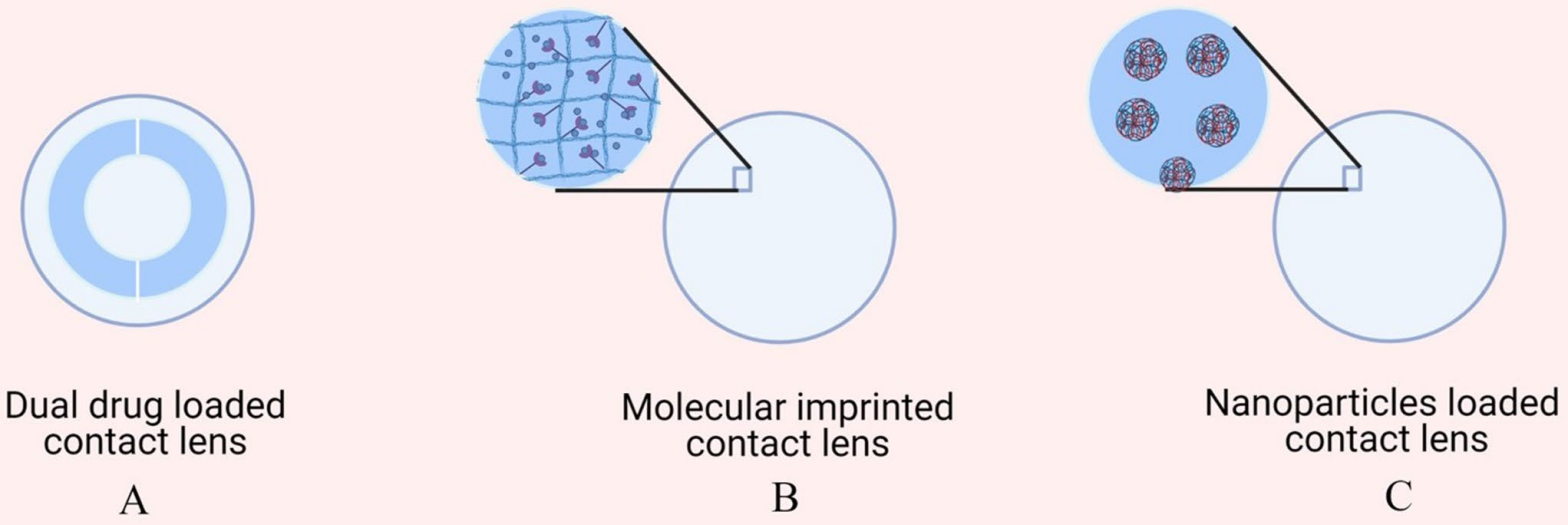
Different types of contact lenses: (**A**) drug-loaded contact lens, (**B**) molecularly imprinted contact lens and (**C**) nanoparticle-loaded contact lens

The idea of drug delivery using contact lenses was first published by Hehl et al. in 1991, wherein the soaking of the contact lens in drug solution was used as a loading method and could sustain the residence of the drug for up to 30 min. Furthermore, various other methods, such as molecular imprinting, implantation of drugs and particle loading of drugs, as well as the application of thermogels for drug loading, have been researched to sustain the release of the drug from contact lenses [95] for up to days to weeks, as illustrated in Fig. 4. The keys to success are being comfortable for a patient to wear for a long period and not causing visual disturbance. 2-Hydroxyethyl methacrylate (pHEMA) and N-vinylpyrrolidone (NVP) are the most often used candidates in the production of soft contact lenses because of their physical and chemical properties. These hydrogels provide high oxygen permeability, sufficient water content and suitable wettability when applied on the ocular surface [96, 97]. Moreover, the nature of the material allows it to conform to the contour of the eyeball [98].

One of the main goals for the development of controlled-release drug delivery systems is to improve drug delivery efficiency. Contact lenses have been designed to overcome the challenge posed by topical formulations to improve patient compliance and minimize undesirable systemic side effects, particularly in chronic disorders such as glaucoma and dry eye syndrome [99]. The ease of application allows patients to self-administer a contact lens similar to other contact lens products available on the market. Furthermore, the development of “bandage contact lenses” with antibiotic or anti-inflammatory therapy for corneal wound healing could provide a better alternative to topical eye drops. Soft contact lenses appear to be perfect candidates for an ocular drug reservoir because they provide a higher bioavailability of drug and offer a considerable dose benefit over ocular drops [99, 100]. However, some drawbacks of a contact lens remain to be considered, such as the chance of infection if it is worn incorrectly and patients experiencing eye discomfort if it is worn long term; therefore, frequent refreshment of the contact lenses may be needed. Another issue is that legal regulations regard contact lenses as medical devices but do not define whether therapeutic contact lenses are drugs, medical devices, or a fusion of both [101]. The difficulty of legal development and regulatory requirements hinder the dynamic speed of development of therapeutic contact lenses [94].

Although numerous publications have reported the application of contact lenses for anterior segment drug delivery to the eye, very few mechanisms for enhancing drug delivery from CLs have been reported. Soaking soft CLs in drug solutions has been one of the most explored methods of drug loading into CLs. However, the major drawbacks of the soaking method are the very high burst release of drugs upon application and the limited duration of drug release, which is often limited to hours to one day [102]. However, there have been several reports of drug delivery via contact lenses, where the affinity of the drug within the CLs is increased by the creation of a high-affinity matrix within the CLs or drug permeation barriers within the CLs to slow its release. Permeation-limiting strategies, such as loading of Vit-E microparticles, drug-loaded liposomes and nanoparticles, have been used to prepare sustained-release CLs, which are further discussed in detail.

Loading different drugs within a single contact lens has been explored as a strategy to address multiple targets in the management of glaucoma. Desai et al. reported the preparation of semicircular rings implanted within contact lenses loaded with timolol and hyaluronic acid for the management of glaucoma. The fabrication of the implant involved the preparation of semicircular rings loaded with the drug, which were fused in a contact lens. The polymers used for the fabrication were photocrosslinked hydroxyethyl methacrylate (HEMA) and ethylene glycol methacrylate (EGMA), along with other additives, to formulate semicircular ring-shaped implants. Under in vitro conditions, the lenses showed high initial burst release (> 85%), followed by a rapid decline within 96 h. The high burst release could be explained by the presence of a high surface drug (timolol). In contrast, the in vivo timolol release lasted for approximately 72 h, although the pharmacodynamic effects lasted for over 144 h in a rabbit model [103]. This study demonstrated the benefits of semicircular ring-implanted contact lenses over conventional soaked CLs (drug release within a day) and conventional timolol maleate eye drops (for a maximum duration of 8 h).

**Fig. 5:**
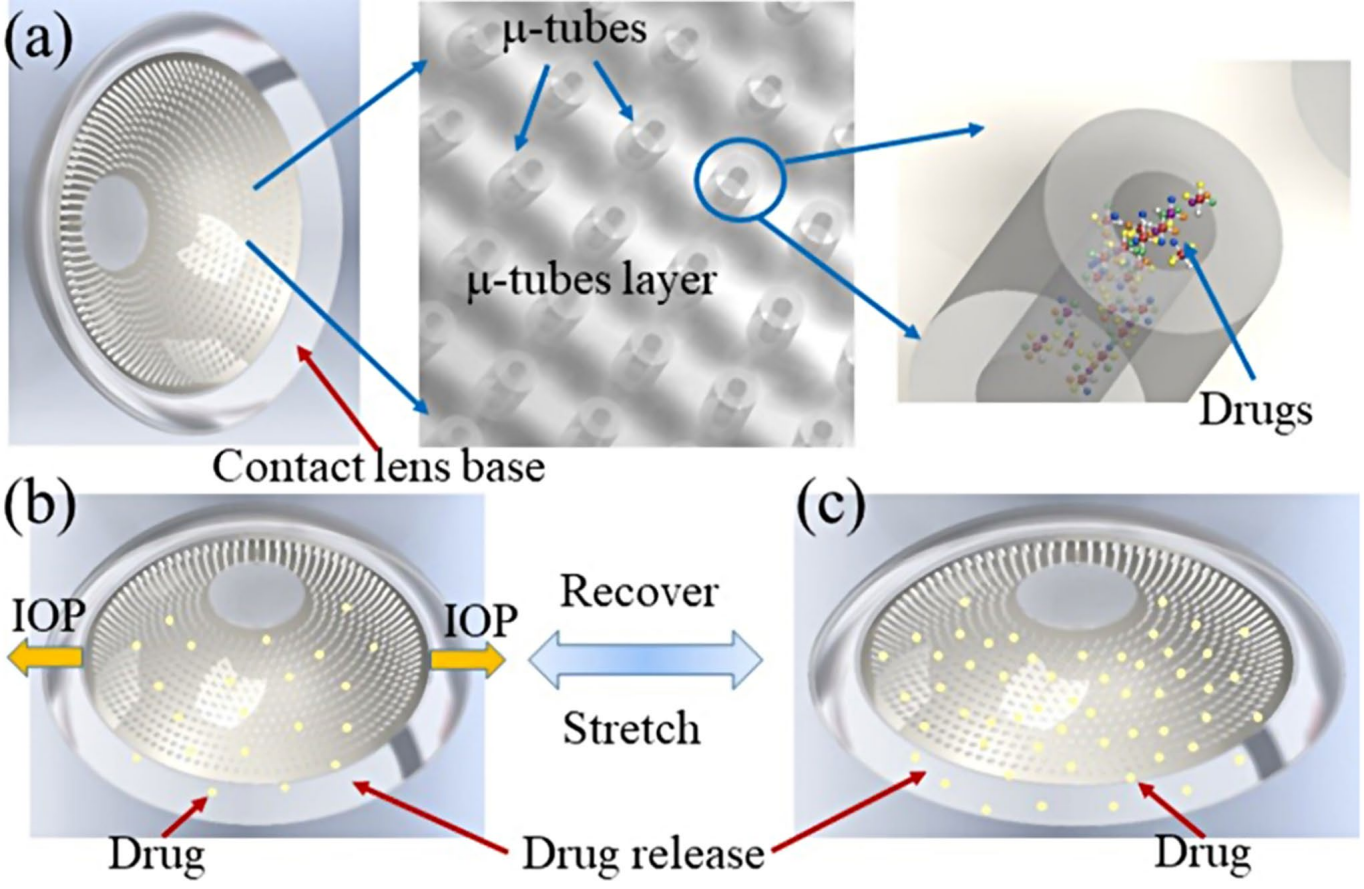
(**a**) A schematic representation of the contact lens featuring embedded microtubes (µ-tubes) serving as drug reservoirs for both diffusion-based and adaptive drug delivery mechanisms. Both the contact lens substrate and the arrayed µ-tubes are constructed from PDMS. (**b**) Depiction of the contact lens device in a neutral state, illustrating diffusion-based drug release. (**c**) Visualization of the contact lens device subjected to mechanical stretching, demonstrating increased drug release from the µ-tubes due to stretch-induced drug delivery [104]

In a recent study, Ding et al. introduced a novel soft contact lens device featuring embedded microtubes for sustained and self-adaptive drug delivery in glaucoma treatment. Through diffusion-based release and IOP-triggered mechanisms, the device achieves prolonged drug release over 45 days with improved bioavailability. Figure 5 shows the device’s self-adaptive capability: as the IOP increases, the contact lens stretches, facilitating enhanced drug release from the microtubes. Conversely, during decreased IOP, drug release diminishes, ensuring optimal dosing [104].

The development and formation of composite drug delivery systems based on layered double hydroxide nanoparticles (LDHs) in thermogels for the management of severe glaucoma was reported by Sun et al. The fabrication of this system involved the preparation of brimonidine-loaded LDH nanoparticles (size 86–100 nm), followed by dispersion in a PLGA–PEG–PLGA copolymer matrix. The sol‒gel transition of a transparent 25% w/w PLGA-PEG-PLGA copolymer matrix at room temperature led to the formation of a hydrogel at approximately 37 °C. The nanoparticles showed a very high burst release of 75% in the first 15 min, followed by sustained release for up to 4 h, whereas the composite system showed a burst release of approximately 10% in the first 30 min, followed by slow release for up to 144 h. Furthermore, the composite thermogel system could sustain the IOP reduction effect for up to 40 h compared with brimonidine eye drops, where the conventional eye drops returned back to the basal IOP, but the composite still resulted in a 2 mmHg IOP reduction [105]. Interestingly, in the in vivo study, external contact lenses were applied on the ocular surface to achieve an even spread of the composite gels to enhance gel retention; however, this approach could interfere with the transmittance of light.

Maulvi et al. fabricated TM-based nanoparticles along with an ethyl cellulose polymer, which were further incorporated into a hydrogel-based contact lens based on HEMA, EGDMA, and methacrylic acid. This CL aimed to use a nanoparticulate system to achieve long-term sustained release of TM. The sizes of the nanoparticles ranged from 261 to 340 nm. The contact lenses showed a high in vitro burst release of up to 35% within the first 6 h, followed by sustained release for up to 168 h. The tear fluid concentration showed a sustained decline in the release of the TM concentration for up to 196 h. In parallel, IOP also decreased, thereby showing a long-acting effect compared with conventional timolol eye drops, which lose the IOP effect within 8 h of application [106].

Kumar et al. explored the utilization of Eudragit nanoparticle-laden contact lenses for prolonged and controlled glaucoma therapy. These lenses were optimized with a specific ratio of Eudragit S100 to polyvinyl alcohol, demonstrating exceptional characteristics, such as a particle size of 102.42 nm ± 15.56 and an entrapment efficiency of 86.99% ± 1.90. Through dynamic light scattering analysis, the authors confirmed the uniform distribution and nanosize of the nanoparticles. Furthermore, in vitro release studies revealed a sustained drug release profile over 12 days, with drug release following zero-order kinetics and Fickian diffusion with swelling identified as the primary mechanism. Ex vivo permeation studies yielded remarkable results, with nanoparticle-loaded lenses achieving over 40–50% greater drug permeation than conventional eye drops, which was attributed to the presence of postlens tear film [107].

#### Hydrogels for topical application

Hydrogels are polymeric crosslinked networks that contain high amounts of water in the polymeric matrix. These polymeric matrices offer various beneficial outcomes for drug delivery by providing spatial and temporal control over the drug release of medicinal agents [108]. The low efficacy of topical eye drops is often the result of rapid clearance following topical instillation. One of the strategies to increase the residence of a drug is the application of a hydrogel matrix for drug application, which reduces drug clearance due to tear turnover caused by increased viscosity. Hydrogels can therefore provide long-term treatment efficacy. Owing to their relatively high degree of ocular residence and sustained drug release, hydrogels are among the most actively researched topics for topical ocular treatment [109]. Various hydrogel-based drug delivery systems for ocular drug delivery are discussed below in detail.

Chitosan-based hydrogels were prepared by Cheng et al. for the topical delivery of latanoprost in glaucoma management. The hydrogel was prepared by mixing a solution of gelatin and chitosan with glycerol phosphate, and latanoprost was added to prepare the final gel. The sol‒gel transition temperature of the gel was 34.18 ± 0.67 °C. Topical delivery of the gel sustained the release of latanoprost for 7 days, with up to 51% release occurring. Compared with the control, the topical gel was able to reduce the IOP via weekly application of the latanoprost-loaded hydrogel. In vivo biocompatibility and efficacy studies following topical application were performed on a weekly basis in a rabbit model. The results suggested a reduction in IOP for 7 days, which remained normal during the course of the study without affecting corneal integrity; moreover, no significant difference in the therapeutic efficacy of the marketed formulation and hydrogel was observed [110].

Li et al. reported the preparation and evaluation of brinzolamide-loaded thermosensitive gels based on poloxamer 407 (F127) and IPR-69 resins by the formation of drug–resin complexes. The amine group of the resin was reacted with the cationic polymer resin to form a gel via ion exchange. The drug–resin complex was further used as an in situ gelling agent for intraocular application. The first-order kinetics of in vitro drug release revealed an initial release of 10%, followed by slow sustained release for up to 8 h, whereas the marketed formulation showed a high burst release of 70% in the first hour of drug release. Furthermore, ocular irritation tests and in vivo tests using a rabbit model suggested that the topical gel has better performance than marketed eye drops in sustaining drug release, as an approximately 30% higher brinzolamide concentration was observed in the aqueous humor post application, although no increase in the duration of action was observed [111].

Taka et al. explored the efficacy of an ac-(RADA)4-CONH2 peptide hydrogel as a carrier for the codelivery of timolol maleate (TM) and brimonidine tartrate (BR) in glaucoma therapy. Rheological studies revealed alterations in gelation kinetics and viscoelastic properties after drug loading, with a significant decrease in hydrogel stiffness attributed to interactions between peptide nanofibres and active compounds. AFM analysis revealed changes in nanofiber length, indicating potential impacts on the hydrogel structure. In vitro release studies demonstrated rapid initial release followed by sustained release of both drugs for up to 24 + hours, highlighting controlled release kinetics. Ex vivo permeability studies revealed significantly greater corneal permeability for TM and BR from the peptide hydrogel than for those from the other drug solutions, suggesting improved drug penetration into the corneal tissue [112].

#### Nanoparticulate drug delivery systems for topical application

Nanoparticulate drug delivery systems such as nanoparticles, nanoemulsions, nanosuspensions, liposomes, niosomes and nanocrystals have been explored as topical ocular drug delivery systems owing to their properties such as mucoadhesion and penetration, as well as sustained drug release. Topical and intraocular drug delivery systems have been developed on the basis of these nanoparticulate systems and are currently being researched for their clinical efficacy [113]. The topical instillation of nanoparticles with various attractive properties, such as mucoadhesiveness, increased drug loading, and enhanced corneal permeability, has been explored for the long-term treatment of glaucoma. Composite formulations of nanoparticle-loaded hydrogels would further benefit long-term glaucoma treatment by offering enhanced corneal retention and sustaining the release of medicinal agents over the period of ocular application [114]. Since nanoparticulate systems often suffer from high burst release of drugs, limiting the duration of action, various strategies, such as the fabrication of lipidic particles and the preparation of polymeric nanoparticles with high affinity for the drug employed, are discussed in detail [115].

Among these systems, liposomes have emerged as a particularly promising drug delivery method for glaucoma treatment. When composed of phospholipid bilayers, liposomes encapsulate drugs, improving their penetration through the corneal barrier and facilitating sustained release. This mechanism allows for the effective combination of multiple medications, such as timolol maleate and brimonidine tartrate, resulting in significant intraocular pressure reduction compared with conventional therapies. Additionally, liposomal formulations protect ocular surfaces from irritation and increase patient compliance, making them valuable tools in the management of glaucoma. Overall, the unique properties of liposomes position them as significant advancements in ocular drug delivery strategies, warranting further research and clinical application [116, 117].

Dorzolamide is a CAI used for the management of glaucoma; however, the bioavailability of conventional dorzolamide eye drops is poor. Hence, dorzolamide-loaded niosomal vesicles were developed by the thin film hydration method and evaluated for ocular delivery by Dehaghi et al. Dorzolamide was loaded using the phosphate gradient method, which is facilitated by the concentration gradient of phosphate buffer during the dialysis stage of fabrication, and the passive loading method, in which dialysis was performed during fabrication. The formulations fabricated via these methods were compared for their drug loading entrapment efficiency and in vitro release profile. The noisome was found to be between 150 and 800 nm in size, with a loading efficiency of up to 25% w/w. The nanoparticles were loaded with d-α-tocopheryl polyethylene glycol 1000 succinate (TPGS) to slow the release of dorzolamide hydrochloride, with the hypothesis that a higher lipid content can aid in the sustained release of the drug because the lipophilic nature of the drug maximizes the drug excipient interaction. The incorporation of TPGS led to a decrease in the cumulative release over 8 h by 15%, suggesting the role of TPGS as a stabilizer of the niosomal bilayers [118].

Shrivastava et al. developed an NLC loaded with two drugs (brinzolamide and timolol maleate), which aims to improve the efficacy of glaucoma therapy by improving the permeation of drugs, enhancing bioavailability, and increasing the precorneal residence time. The two drugs were encapsulated within sesame oil, while Tween 80 and Precirol ATO 5 formed the shell layer via a melt emulsification technique. The physicochemical properties of NLCs, such as drug loading (DL), entrapment efficiency (EE), in vitro release and ex vivo penetration, were evaluated. The DL and EE of the optimized NLC formulations were 0.71 ± 0.02% and 70.73 ± 0.64% for brinzolamide and 0.360 ± 0.01% and 77.12 ± 0.64% for timolol maleate, respectively. The in vitro release profiles of the dual drugs were relatively similar; the burst release rates within 5 h were 38 ± 3.10% and 34 ± 2.90% for brinzolamide and timolol maleate, respectively, whereas 70.08 ± 6.40% and 72.29 ± 5.90% of the burst release rates were released after 24 h for brinzolamide and timolol maleate, respectively. An ex vivo penetration study demonstrated that 36.20 ± 2.80% of brinzolamide and 33.47 ± 2.80% of timolol maleate permeated in the initial 5 h and then progressed to 67.69 ± 6.500% and 72.30 ± 6.40% in 24 h, respectively. The release patterns and permeation of both drugs from NLCs were significantly greater than those from their suspension formulations [119].

Galactosylated chitosan-based nanoparticles (GC-NPs) were developed by Zhao et al. for the delivery of timolol maleate. The size of the nanoparticles was 213.3 ± 6.83 nm, and the zeta potential was 30.2 ± 0.46 mV. The drug loading efficiency was 17.72 ± 0.28%, and the entrapment efficiency was 38.58 ± 1.31%. The egg chorioallantoic membrane assay confirmed that the formulation was nonirritant to ocular tissues. Both the in vitro release and in vivo pharmacodynamic studies suggested that the optimized nanoparticles have a longer action time and greater efficacy in reducing IOP than the marketed topical timolol maleate eye drops do. The conventional timolol maleate eye drops had a maximum IOP reduction of 6 mmHg in 4 h and lasted up to 8 h, whereas the timolol maleate GC-NPs showed a peak IOP reduction of 10 mmHg for an extended duration of action of 12 h, i.e., 4 h more than the marketed formulation [120].

**Fig. 6:**
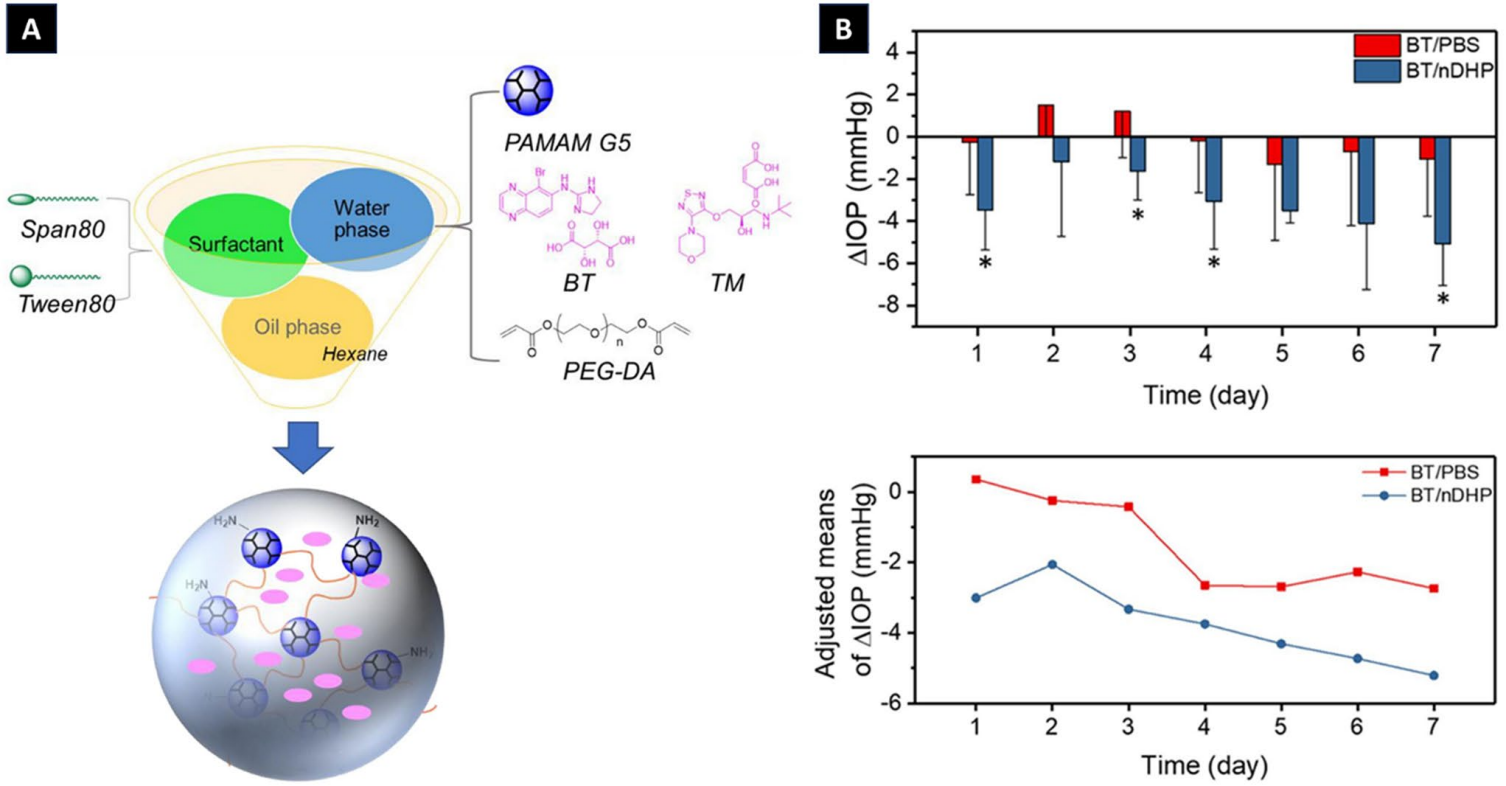
(**A**) Schematic illustration of the preparation of nDHPs. (**B**) In vivo IOP-lowering effect of nDHP in normotensive rats after 7 days of daily topical administration [121]

In a different study, Wang et al. reported the fabrication of nanoin-nano dendrimer gel particles (nDHPs) for enhancing the topical delivery of antiglaucoma drugs. Leveraging the inverse emulsion aza-Michael addition method, they successfully downscaled dendrimer hydrogel particles (DHPs) into their nanostructured counterparts with precise control over the particle size and surface characteristics. Compared with micron-sized DHPs, nDHPs exhibit superior cytocompatibility, corneal permeability, and drug release kinetics. Pharmacokinetic assessments revealed that nDHPs have sustained and potent IOP-lowering effects in both normotensive and genetic glaucoma models (Fig. 6). Importantly, nDHPs facilitate the permeation of combined drugs while offering high drug encapsulation capacity and programmable release profiles, which are essential for tailored therapeutic interventions [121].

Table 3 below summarizes key findings from various recent studies on novel LADDS for glaucoma treatment. This review highlights a diverse range of innovative approaches, including drug-loaded contact lenses, nanoparticle-based systems, hydrogels, and liposomes. These LADDSs offer unique advantages in terms of sustained drug release and improved bioavailability, with durations of action ranging from hours to several weeks. The systems aim to address challenges associated with traditional eye drop formulations, such as poor patient compliance and limited drug bioavailability. By providing long-to-ultralong therapeutic effects and reducing dosing frequency, LADDSs have the potential to significantly improve treatment efficacy in glaucoma management. This overview underscores the importance of continued research and development in this field to enhance patient outcomes and quality of life.

**Table 3:** Summary of novel LADDSs for glaucoma treatment explored in recent years

LADDS Type	Key features	Drug(s)	Duration of action	Notable findings	Materials used	Route of application	References
Contact Lenses	Drug-loaded, molecularly imprinted, nanoparticle-loaded	Timolol, Hyaluronic acid	Up to 144 h	High initial burst release, but longer-lasting effects than eye drops	pHEMA, NVP, HEMA, EGMA	Topical	[122]
Microtube-embedded Contact Lenses	Self-adaptive, IOP-triggered release	Not specified	Up to 45 days	Improved bioavailability, adaptive release based on IOP	PDMS	Topical	[123]
LDH Nanoparticles in Thermogels	Composite system	Brimonidine	Up to 144 h	Sustained IOP reduction for up to 40 h	PLGA-PEG-PLGA copolymer	Intravitreal	[124]
Nanoparticle-laden Contact Lenses	Eudragit-based	Not specified	Up to 12 days	40–50% greater drug permeation than eye drops	Eudragit S100, polyvinyl alcohol	Topical	[125]
Chitosan-based Hydrogels	Topical application	Latanoprost	7 days	Comparable efficacy to marketed formulation	Chitosan, gelatin, glycerol phosphate	Topical	[126]
Peptide Hydrogel	Codelivery system	Timolol maleate, Brimonidine tartrate	24 + hours	Enhanced corneal permeability	ac-(RADA)4-CONH2 peptide	Topical	[112]
Liposomes	Phospholipid bilayers	Various (e.g., Timolol maleate, Brimonidine tartrate)	Not specified	Improved penetration, sustained release, reduced irritation	Phospholipids	Topical	[127]
Liposomes	encapsulation of prostaglandin derivatives for sustained IOP reduction	Latanoprost	Up to 120 days	Improved drug stability and sustained release	Unilamellar vesicles, phospholipids	Subconjunctival injection	[128]
Polymeric Hydrogel Microspheres	Microsphere-based sustained release technology	Timolol maleate, Brimonidine tartrate	Up to 107 days (Timolol), Up to 28 days (Brimonidine)	Long-acting, noninvasive application, SoilDrop technology	PLGA, PLA	Topical	[129, 130]

### Periocular drug delivery systems

Topical application of LADDS improves patient acceptance, but the therapeutic benefits are often limited owing to the presence of various static and dynamic ocular barriers. The most obvious target for the treatment of glaucoma is the supraciliary and suprachoroidal space of the eye; multiple studies have demonstrated reliable and effective drug delivery to these spaces, with no major safety concerns in either human subjects or animal models [131–134]. However, few antiglaucoma-loaded LADDS have been developed for delivery via this route. Alternatively, less invasive approaches, such as the subconjunctival route, have been widely researched for glaucoma treatment.

#### Supraciliary/supracoroidal route

Targeted delivery to the supraciliary space via hollow microneedles has been tested using less concentrated solutions of antiglaucoma drugs (sulprostone and brimonidine). The results showed that targeted injection via microneedles had similar IOP-lowering effects to those of highly concentrated topical eye drops, and both methods reduced IOP by 3 mmHg for 9 h. In this study, targeted delivery to the supraciliary space by microneedles needed a 100-fold lower dose than topical administration to achieve a similar pharmacodynamic response [131]. In light of suprachoroidal space delivery, a study was carried out using a two-component drug particle and hydrogel carrier. The device aimed to deliver the model drug to the posterior region of the suprachoroidal space *by pushing* it by hydrogel swelling. Although researchers have used 1% hyaluronic acid as a model compound, the design of this device suggests potential applicability for delivering antiglaucoma drugs with similar physicochemical properties. One drawback of this delivery system is that the IOP rapidly decreases to 4 mmHg after microneedle injection and then gradually recovers to the normal IOP 6 days after injection. The factors contributing to the decrease in IOP may include the inflammatory response, increased uveoscleral outflow, or pushing of a hydrogel that blocks the ciliary body, hence reducing aqueous humor secretion [135]. Similarly, Chiang et al. developed poly(lactic acid)-based brimonidine-loaded microspheres to sustain release for one month via a single injection. The formulation was injected through the sclera via hollow microneedle devices to target the supraciliary space. Glaucomatous rabbit models were used for in vivo testing. The IOP of the animals initially decreased by 6 mmHg, and brimonidine release was sustained for 35 days. All the animals tolerated the treatment well, without significant side effects, i.e., ocular irritation or painful sensations after the administration of a microneedle [136], and tolerability was measured in the suprachoroidal injection of triamcinolone acetonide study by Goldstein et al. [137].

#### Subconjunctival route

A novel ocular insert composed of ethyl cellulose and the natural polymer sodium alginate was applied in the cul-de-sac to deliver TM. The hydrophilic polymers integrated into a hydrophobic matrix system, and thereby, the surface of the ocular insert was easily wetted by tear fluids. Hence, such a matrix system facilitated the constant and sustained release of TM to the site of application. The physicochemical properties of this ocular insert were characterized, and it showed greater than 90% content uniformity and drug entrapment efficiency. The in vitro drug release reached 72.1% after 9 h. Ex vivo permeation tests proved that the choice of polymer type affects the permeability coefficient. The in vivo results revealed a significant reduction in IOP (from 30 mmHg to 14 mmHg) in rabbits treated with the inserts within three days compared with those in rabbits treated with marketed eye drops (from 30 mmHg to 27 mmHg), as measured by a tonometer. Furthermore, ocular safety testing and insert stability studies were performed in accordance with ICH guidelines. This TM-loaded ocular insert can be cast into various shapes fit into the upper and lower conjunctival sacs for action. This study demonstrated that TM-loaded ocular inserts could be an alternative to conventional eye drops for the long-term treatment of glaucoma [138].

Chitosan/hydroxyethyl cellulose-based ocular inserts were developed for the sustained release of dorzolamide. Dorzolamide inserts (1 × 2 mm) were fabricated via the casting/solvent method, and their physicochemical properties were characterized. Ex vivo biodistribution and gamma scintigraphy were evaluated in pharmacokinetics studies. The efficacy of the inserts was tested in glaucomatous rats. The hydrophilic drug strongly interacted with the chitosan/hydroxyethyl cellulose matrix, but the in vitro results revealed that 75% of the drug was released within 3 h. Ex vivo biodistribution studies and scintigraphy images revealed that more than 50% of 99mTc-dorzolamide remained in the eye 18 h postinsert administration, whereas approximately one-quarter of the drug remained in the eye when dorzolamide eye drops were used. The inserts were reported to exert constant hypotensive effects for 14 days after a single administration, which was equivalent to 3 times per day to maintain treatment efficacy. This report suggested that the potential application of LADDS with dorzolamide-loaded inserts can be beneficial in glaucoma treatment by preventing further RGC death [139].

A core‒shell polymer microsphere-based LADDS with brimonidine tartrate (BT) was developed by Veloso et al. [140]. Microspheres act as reservoirs of BT and deliver it to the targeted site by diffusion via the subconjunctival route. In this study, the microspheres consisted of poly(d, l-lactic-co-glycolic acid) as the core and poly(l-lactic acid) (PLLA) as the shell, which released BT over 40 days via diffusion through the PLLA-rich shell. In vivo studies were carried out in glaucomatous rabbits, and the results demonstrated an immediate and significant IOP reduction of 20 mmHg in rabbit models, where the IOP-lowering effect lasted for 55 days [141]. The results revealed that BT-loaded microspheres were noticeably superior to the 2–6 mmHg reduction usually attained from multiple daily administrations of conventional alphagan eye drops. BT-loaded microspheres developed by Ying et al. could provide long-term and steady delivery of BT to the subconjunctival space and overcome the challenge of the short, 3-h half-life of alphagan eye drops.

Natarajan et al. reported the fabrication of latanoprost-loaded nanostructured lipid carriers (NLCs) and demonstrated a sustained reduction in IOP in glaucomatous nonhuman primate models. The carrier used for the loading of the drug was nanosized unilamellar vesicles. The properties of NLCs have been elucidated via various physicochemical techniques, including isothermal titration calorimetry, dynamic light scattering and cryo-TEM. The results from the latter showed that the spherical shape of the liposomes was maintained even at higher drug loading rates because of some specific molecular interactions between the lipid and the drug. This finding also explains why drug-loaded NLCs have improved sustained release and stability properties. In an in vivo study, the results were comparable between latanoprost-loaded NLCs and topical latanoprost eye drops that were applied once daily; latanoprost-loaded NLCs and latanoprost-loaded NLCs demonstrated long-term efficacy in reducing IOP for 120 days [142].

Acetazolamide (ACZ)-loaded poly(propylene imine) dendrimer nanoarchitectures were developed by Mishra et al. as potential IOP-lowering topical formulations. ACZ is currently available as a modified-release tablet for the treatment of all forms of glaucoma, but its lack of specificity for targeting induces many systemic side effects, such as hemorrhage, metabolic acidosis or skin rashes. ACZ-loaded dendrimers were designed to carry and release ACZ to specific ocular tissues with longer ocular residence times and minimal systemic side effects, all of which hopefully would improve patient compliance with the treatment. Optimized ACZ formulations were evaluated for ocular tolerance, hemolytic toxicity, and IOP-lowering effects in normotensive New Zealand albino rabbits. The entrapment efficiency was found to be approximately 56 ± 2.3%, while in vitro release studies revealed the sustained release of over 80% of 25 µM ACZ in 24 h. A longer residence time of ACZ in the cul-de-sac was found when the drug was encapsulated in dendrimer nanoarchitectures, resulting in prolonged and sustained release. Furthermore, ACZ loading resulted in a greater reduction in IOP than did systemic ACZ loading, which suppressed IOP by 7 mmHg compared with 4 mmHg after 4 h of administration. However, the ocular tolerance test, a modified Draize test, suggested that the formulations at 10 and 25 µM were weakly irritant to the eyes, whereas the control solutions did not cause any eye irritation or watering reflux in the rabbit models. As a result, poly(propylene imine) dendrimers have improved ocular drug retention time, are more effective at reducing IOP, and are safe and effective for the treatment of glaucoma [143].

A novel NO-releasing subconjunctival system composed of mesoporous silica nanoparticles delivers sodium nitroprusside, an NO-donating drug, to Schlemm’s canal and trabecular meshwork. The results revealed that mesoporous silica nanoparticles (MSNs) loaded with sodium nitroprusside significantly reduced the IOP for a longer duration (from 3 to 48 h) in mouse models, with results comparable to those with a 1/40 dose of SN solution. In this study, MSNs demonstrated efficient penetration ability for bypassing the transendothelial electrical resistance of the cell monolayer on the cornea and delivering sodium nitroprusside particles directly to Schlemm’s canal and the trabecular meshwork. The sustained release of NO facilitated by MSNs provided a longer and more effective IOP reduction duration of up to 48 h [144]. Another novel long-acting NO donor system releases polydiazeniumdiolate (NOP) and is composed of carbon-bound polydiazeniumdiolate. It targets the conventional outflow pathway to lower the IOP. The NO release level was closely monitored via real-time detection of gas, along with analysis of the accumulated release profile. On the basis of the experimental results, 100 µM NO was released from 50 µg/mL NOP, which resulted in optimal trabecular meshwork relaxation in the gel contraction assay. On the other hand, 350 µM NO was delivered locally by 100 µg/mL NOP for over 48 h and effectively lowered IOP in vivo without side effects in normotensive rabbit models [145].

### Intraocular drug delivery systems

Compared with topical routes and periocular routes, intraocular drug delivery systems, i.e., intracameral or intravitreal routes, are known to be the most invasive routes for administering LADDS to the eye. Despite the invasiveness of these routes, more so with the intracameral route, the drug is delivered directly to the target site. Formulations such as particles, gels, preformed implants, and devices can be administered via this route to achieve the sustained release of therapeutics. The following sections detail several LADDSs developed recently that target the treatment of glaucoma.

#### Intracameral route

**Fig. 7:**
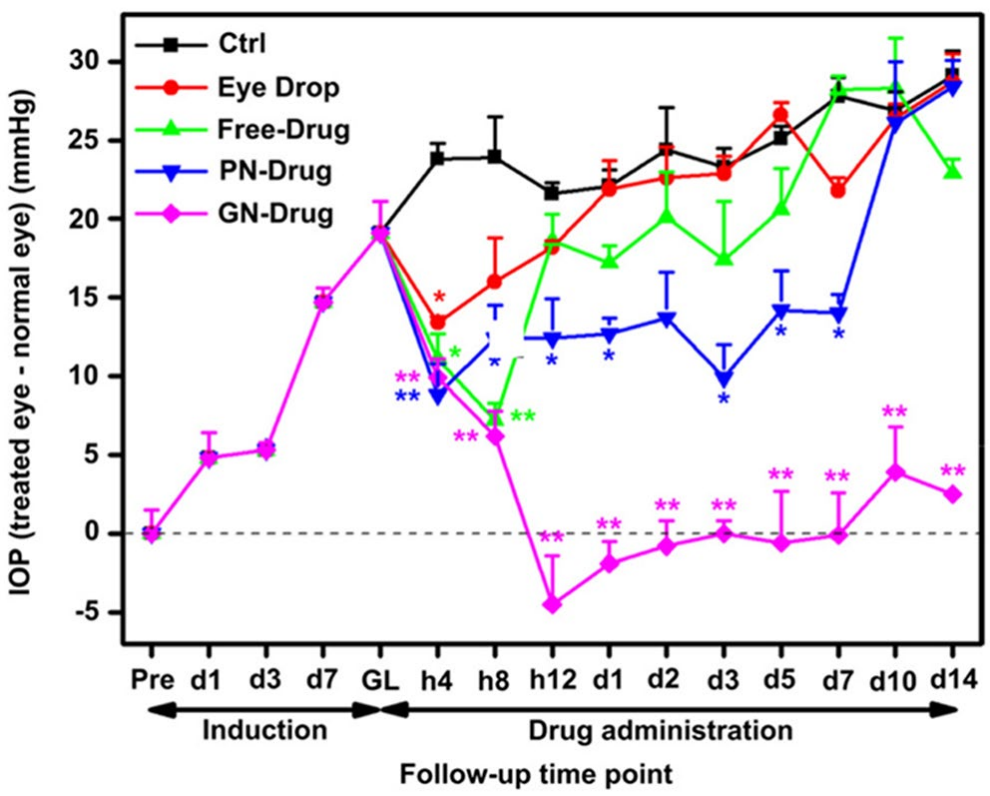
Reduction in IOP following the administration of various pilocarpine drug delivery systems [146]

Biocompatible and biodegradable hydrogels are ideal carriers in the design of LADDSs. For in situ ocular applications, a hydrogel must transit into sol‒gel form from the liquid form after the delivery system is administered [108]. Different forms of responsive polymeric drug delivery systems have been studied for ocular application, as illustrated in Fig. 7. A biodegradable thermoresponsive in situ intracameral delivery system consisting of grafted gelatin with a carboxylic end-capped poly(N-isopropylacrylamide) was fabricated via a carbodiimide-mediated coupling reaction, and this thermoresponsive hydrogel was used for loading pilocarpine as a model drug [146]. The drug encapsulation efficiency of the hydrogel was 55–60%. These results suggested that a greater drug payload led to faster gelation of the copolymers. A loading efficiency of up to 60% was achieved with the graft polymers, and the in vitro drug release data suggested that the grafted polymer was able to sustain the release of pilocarpine for up to 14 days following an approximately 60% burst release on the first day. The in vivo studies were performed using rabbit models, and the drug release in the aqueous humor was found to be significantly greater in the intracameral injected graft polymer. The hydrogel was also able to significantly lower the IOP in a rabbit glaucoma model following a 50 µL injection of graft polymer containing 2% drug.

Chou et al. investigated the intracameral injection of pilocarpine-loaded thermoresponsive gels. The authors reported in vivo evaluation of gallic acid (GA)-grafted gelatin-g-poly(N-isopropylacrylamide) (GN) polymer hydrogels for the delivery of pilocarpine. The gelatin was functionalized with ascorbic acid, which was then grafted with GA via a redox radical initiation reaction. GA and ascorbic acid are used as antioxidants to reduce the excessive oxidative stress that causes glaucomatous eyes. Furthermore, pilocarpine was loaded into the thermogel for sustained ocular delivery following intracameral administration. More GA conjugation was able to decrease the burst release, and optimum conjugation could maintain the release of pilocarpine for up to 28 days under in vivo conditions. As a result, this thermogel was proven to significantly lower the IOP in glaucomatous rabbit models driven by an antioxidant polymeric drug delivery system [147].

An intracameral device was developed using thin polycaprolactone (PCL) films as carriers and loaded with a novel hypotensive drug, DE-117, and its hydrolysed form hDE-117 via the spin-casting technique. The dimensions of this intracameral device ranged from 2 to 3 mm and were made of 45-µm-thick PCL films. The device was then loaded with drugs and sealed via nichrome loops. This device delivered DE-117 with zero-order release kinetics for approximately six months, as confirmed by in vitro release studies. For in vivo studies, rabbit models were used, and the results revealed the IOP-lowering effect of the devices over 24 weeks. Drug distribution within the ocular tissue after 5, 12 and 24 weeks of implantation suggested that more drug was present in the anterior chamber, followed by very low concentrations in the vitreous humor. DE-117 was released at a rate of 0.49 µg per day for up to 180 days, whereas hDE-117 was not detected, which confirmed that DE-117 was protected from hydrolysis within the device. In terms of histological studies, only one model eye developed transient subconjunctival hemorrhage out of 16. This prototype intracameral implant demonstrated controlled release kinetics, as both DE-117 and hDE-117 could still be detected in the targeted tissues and aqueous humor for over 24 weeks. The in vivo results from normotensive rabbit models revealed that the IOP decreased to 7.1 ± 1.8 mmHg from baseline after the first week of implantation, whereas the IOP of the contralateral eye was slightly reduced to 0.3 ± 2.9 mmHg, which was similar and fluctuating throughout the study. This intracameral device still requires further optimization in terms of shape and dimensions and has yet to be tested in larger animals [148].

Nguyen et al. investigated the impact of shell thickness in hollow poly(lactic acid) nanoparticles (HPLA NPs) on sustained drug delivery for glaucoma treatment, revealing critical insights for nanoparticle-based ocular therapy. Through comprehensive in vitro and in vivo assessments, they revealed that pilocarpine-loaded HPLA NPs exhibited exceptional biocompatibility with ocular tissues, even at high doses. Notably, HPLA NPs with a 40-nm-thick shell demonstrated optimal sustained drug release, maintaining therapeutic levels for more than 56 days, outperforming their counterparts with thinner or thicker shells. Moreover, these 40-nm-thick HPLA NPs show superior efficacy in lowering intraocular pressure, preserving ocular structures, and inhibiting disease progression in glaucoma models. Their study underscores the pivotal role of shell thickness in nanoparticle design, offering promising avenues for developing long-acting drug delivery systems for chronic ocular diseases such as glaucoma [149].

#### Intravitreal route

The intravitreal route is commonly used for the treatment of posterior segment ocular diseases. Despite the administration process being potentially invasive, this route increases the ocular bioavailability of APIs by overcoming some typical ocular drug delivery challenges, such as low residence time, rapid drainage of a formulation, and noncompliance of a patient. The mechanism of glaucoma treatment in the posterior segment could differ from that in topical and periocular LADDS in terms of lowering IOP. Drugs loaded in intravitreal LADDSs focus mainly on providing neuroprotection to RGCs, whereas topical APIs or drugs loaded in periocular LADDs focus on modifying the physicomechanical properties of ocular tissues to reduce IOP, i.e., increasing drainage of periocular fluid and relaxing the tissues to prevent the buildup of IOP.

A study was carried out by Zou et al. to investigate intravitreal tissue responses using four types of nanocarriers in rabbits. The carriers were made of polystyrene (PS), poly(N-isopropyl acrylamide) (PNIPAM), poly(L-lactic acid) (PLLA), and hyaluronic acid (HA). The intravitreal injection of PS, PNIPAM and PLLA nanoparticles significantly reduced the IOP, with mean reductions of 6, 4 and 3 (± 2) mmHg, respectively, which lasted for less than three days. Surprisingly, the injection of HA nanoparticles had limited IOP-lowering effects. Although Zou et al. could not explain why HA-based nanoparticles did not impact IOP, it was likely that the material properties of the nanoparticles affected ocular compatibility. This study revealed that the injection of nanoparticles had no apparent influence on the anatomical structure or thickness of retinal tissues. On the basis of the morphological assessment of tissue thickness, all the nanoparticles also had no apparent influence on the anatomical structures of the iris or corneal tissue, except for the PNIPAM-treated animals, who presented statistically insignificantly lower thicknesses of the iris and corneal tissue [150].

A study carried out by García-Caballero et al. reported that the sustained delivery of glial cell line-derived neurotrophic factor via poly-lactic-co-glycolic acid/vitamin E-based (GDNF/VitE) microspheres can prevent the death of RGCs. GDNF/VitE microspheres were prepared via a solid‒oil‒water emulsion‒solvent evaporation technique and then injected intravittruly into glaucomatous rabbits. The study lasted for 24 weeks, during which IOP was measured before and at 24 h and then at 1, 4, 6, 8, 12, 18 and 24 weeks postinjection. The eyes were enucleated at different time intervals. The in vivo results revealed that a single administration of GDNF/VitE microspheres developed by Herrero-Vanrell’s group can provide sustained release of GDNF/VitE for up to six months. However, the level of GDNF in the in vivo models could not be quantified in this group, and the eyes remained within the normal IOP limits of 14 mmHg after administration, with no abnormalities of the eye found at the end of the study [151].

A study carried out by Patel et al. demonstrated that they could achieve the sustained release of axitinib and timolol by utilizing a monolithic implant design based on ethylene-vinyl acetate (EVA). They successfully blended axitinib with 50% loading and shaped it into a cylindrical rod-shaped implant. The formulation with 50% axitinib achieved a cumulative release of 57 µg within a 28-day period. Moreover, the formulations with 50% axitinib loading released timolol at a higher concentration than did the formulations with 40% and 10% axitinib loading. In summary, this implant provides matrix drug release, and its design can be adjusted to fit different implantation locations in the eye. The demonstrated one-month release rate, in conjunction with modelling, provides strong evidence that an EVA implant can effectively deliver axitinib for more than six months [152].

Hydrogen sulfide (H2S) has been reported to be beneficial in controlling the progression of glaucoma by providing retinal neuroprotection and reducing IOP. Although H2S was suggested to be a potential candidate for glaucoma treatment, its donors were not stable in water and could be toxic at concentrations greater than 200 µM. Patil et al. developed a nonaqueous intravitreal in situ gelling system for H2S donors. The polypeptide-coglycolide-based delivery system was composed of GYY 4137, an H2S donor, where the PLGA was previously dissolved in a mixture of benzyl benzoate and benzyl alcohol at a ratio of 3:7. The GYY 4137 formulation was physiochemically characterized. Rheological data proved that the formulation can be loaded into a syringe with a viscosity of 50 cP and injected via a 25-gauge needle without causing discomfort to a patient. The GYY 4137 formulation did not significantly affect the Y79 retinoblastoma cell line. Although the group proposed the use of an H2S-loaded in situ gelling system to reduce IOP for the treatment of glaucoma, no in vivo studies have been carried out, as the delivery system became unstable due to the oxidative degradation of H2S after 72 h. Further studies should focus on the modification of the release media and stimulated tear fluid to quantify the concentration of H2S for an extended period [153, 154].

## FDA-approved products and ongoing clinical trials

Owing to its enormous potential in the management of ocular diseases, including glaucoma, LADDS has been and is being developed by various pharmaceutical companies as well as research groups. Although very few have received FDA approval, several such delivery systems are in different stages of clinical trials.

Ocusert, introduced by Alza Corporation, USA, was the first longer-acting ocular drug delivery system approved by the US FDA in the early 1970s. Ocusert is essentially a nondegradable reservoir system containing pilocarpine as an active agent. This ring-shaped reservoir system was made up of a thin ethylene-vinyl acetate microporous membrane supported by a titanium dioxide ring [155]. The system was designed to be placed in the inferior fornix, where it releases pilocarpine at a rate of 20 to 40 µg/hour for 1 week [156]. Although Ocusert was approved by the FDA and launched on the market, it was later discontinued because of side effects such as dislodging of the ring and dose dumping due to initial high burst release, among others [157].

DURYSTA™ is the latest and first intracameral sustained release implant developed by Allergan Plc for the management of OAG. The microimplant is based on Allergan’s biodegradable NOVADUR platform, which consists of a drug extruded in a PLGA copolymer mixture that releases bimatoprost over a 4–6-month period [158, 159]. In clinical trials, the implants were evaluated for 4 different doses of bimatoprost viz. 6, 10, 15 and 20 µg, and it was found that bimatoprost implants controlled the increase in IOP in 40% of enrolled patients for 12 months, whereas it was controlled in another 28% of patients for up to 24 months [160, 161]. In phase III trials involving 528 patients with OAG and ocular hypertension (OHT), the bimatoprost implant group presented a 30% reduction in IOP from baseline in a 12-week period [158]. Among the 4 different dose groups, 10 µg bimatoprost implants were found to be safest and thus were submitted for FDA approval and introduced under the brand name DURYSTA™. Although 82.9% of patients expressed interest in undergoing another implant procedure after 24 months of trial [161], its swelling and slow biodegradation could lead to limitations in widespread use. For example, in a phase I/II study, implants swelled up to 125% of their initial size in some patients and were reduced to ≤ 25% of their initial size by 12 months. This will limit the introduction of another implant in the intracameral space because of space restrictions and possible adverse reactions. Additionally, PLGA copolymer-based implants degrade primarily by a bulk erosion process and may breakdown into several fragments. These fragments may block natural aqueous outflow from the anterior chamber and further increase the IOP.

There are several other promising sustained-release drug delivery platforms, such as the Bimatoprost Ocular ring (Allergan Plc), Topical Ophthalmic Drug Delivery Device (TODD, Amorphex Therapeutics), Evolute^®^ (Mati Therapeutics), Eye-D VS-101 (BioLight Life Sciences), ENV515 (Travoprost XR, Envisia Therapeutics), iDose (Glaucos), and OTX-TIC (Ocular Therapeutics), which are currently being evaluated in different stages of clinical trials.

The Bimatoprost ocular ring is a nonbiodegradable preservative-free silicone matrix ring containing 13 mg of Bimatoprost inside the inner polypropylene ring [158]. This flexible implantable ring, which is 24 to 29 mm in size, is placed into the upper and lower fornix. A phase II clinical trial involving 130 patients with OAG and OHT reported a consistent decrease in IOP of 20% for up to 180 days, but OAG and OHT were found to be less effective than timolol eye drops administered twice daily. On the downside, a total of 28 dislodgements of the implant ring were reported in 15 enrolled patients, while 45.3% treatment-related adverse events were reported [162]. There are no further updates available on this platform since a fixed combination of bimatoprost and timolol ring studies was completed in 2018 (clinical trial number: NCT02742649).

Another ocular insert system, the Topical Ophthalmic Drug Delivery Device (TODDD™) developed by Amorphex Therapeutics, sits on the sclera in the upper eyelid region to deliver a nondrug vehicle from a soft polymer matrix depot over several months. The objective of this study was to investigate whether this large device has comfort issues in the long term. An open-label safety and tolerability study conducted on 14 adult humans reported a 70% retention rate of the prototype after four weeks of continuous wear [163]. The results suggested that this TODDD™ depot could provide a safe and comfortable solution for delivering drugs on site over a longer period.

In the category of punctal plugs, Novelion Therapeutics, Inc., in association with Mati Therapeutics, has developed sustained-release Evolute^®^ punctal plugs for the management of glaucoma and OHT. Mati therapeutics have recently purchased all rights related to the Evolute^®^ Punctal Plug Delivery System (PPDS) from Novelion Therapeutics, Inc [164]. Evolute^®^ is an L-shaped plug consisting of an inner nonbiodegradable drug-eluting core loaded with latanoprost. The plugs can be placed in upper or lower puncta with the help of a slit lamp. Multicenter separate phase II clinical trials conducted on 134 subjects revealed promising decreases in IOP from baseline by 5.7 mmHg over a 4-week period. Additionally, the retention rates of punctal plugs over a 12-week period were 92–96% and 48–58% when the plugs were placed in lower and upper puncta, respectively [165]. Although the retention of punctal plugs is a major challenge, the promising results from these trials provide much hope for the sustained release of PPDS.

The subconjunctival space is an attractive region of interest for the delivery of sustained-release drug delivery systems. A novel platform known as the Eye-D latanoprost insert developed by BioLight Life Sciences was tested on 77 patients in a phase I/IIa study. The Eye-D inserts were placed in the subconjunctival space in an in-office procedure and resulted in an average 24% reduction in IOP over a 12-week period. This proof-of-concept trial also achieved safety and efficacy endpoints [158].

The Envisia Therapeutics ENV515 (Travoprost XR) is an intracameral biodegradable implant containing the antiglaucoma drug travoprost and is manufactured via the company’s PRINT (nanoparticle replication engineering) technology. Compared with daily topical Travatan Z drops, the phase IIa study conducted on 21 glaucoma patients achieved a primary efficacy endpoint. A 12-month clinical study was subsequently conducted on OAG patients, and the fellow eye was treated with 0.5% timolol ophthalmic solution once daily topically. ENV515 implants resulted in a 25% reduction in IOP and were noninferior to timolol-treated eyes over an 11-month period [158, 166].

Another implant system developed by Glaucos for the management of glaucoma is a nondegradable titanium-based traveloprost with an intracameral implant that is anchored to the trabecular meshwork through a small corneal incision. The implant system is called the iDose. Once depleted, the iDose implant needs to be removed and replaced with a new implant. In a multicenter, randomized, double-blinded phase II clinical safety and efficacy study, single administration of fast-eluting iDose implants resulted in a 33% reduction in IOP compared with a 30% reduction in IOP with a daily dose of 0.5% timolol solution over 12 weeks [158]. A 154-subject, multicenter, randomized, double-blind phase IIb trial revealed a substantial reduction in IOP in a 24-month interim analysis of a 36-month phase IIb study. The study also demonstrated the safety profile of iDose implants with no clinically significant corneal endothelial cell loss, no serious corneal adverse events, and no adverse events of conjunctival hyperemia for a 24-month duration [167].

Ocular Therapeutix is another ophthalmic company working on an OTX-TIC intracameral resorbable implant platform for the sustained delivery of travoprost. Micronized travoprost is released from the implant over a period of 4–6 months. The first cohort study of 5 patients revealed a significant reduction in IOP and evidence of biodegradation of the implant by 4 months [168]. In phase I, a prospective, multicenter, open-label, dose escalation clinical trial of OTX-TIC implants, safety, efficacy, durability, and tolerability for up to 18 months was established. Additionally, no serious adverse effects were reported [169].

The narrative highlighted in Table 4 below summarizes key information from each study, including the drug delivery system used, product name, developer, study design, and key findings. This highlights the importance of sustained-release drug delivery systems in glaucoma treatment and emphasizes the need for further research in this area.

**Table 4:** Summary of clinical studies on sustained-release drug delivery systems for glaucoma treatment

Delivery system	Product name	Developer	NCT number (Status)	Drug	Overview
External ocular inserts	Ocular Ring	Allergan, Dublin, Ireland	NCT02742649 (Phase 1/2, completed)	Bimatoprost/ Timolol	• Route: Peri-ocular ring (conjunctival cul-de-sac) • The study examines the effectiveness and safety of a fixed combination bimatoprost/timolol ocular insert compared to its separate components in individuals with open-angle glaucoma or ocular hypertension. It is a Phase 1B trial, multicenter, randomized, double-masked, and controlled, with a crossover to timolol 0.5%.
Punctal plug	Evolute	Mati Therapeutics, Austin, TX, USA	NCT03318146, NCT02014142 (Phase 2, completed)	Latanoprost/ Travoprost	• Route: Punctum • The study is a prospective, open-label, controlled, nonrandomized trial utilizing a comparative “split-body” design. It compares the efficacy of unilateral Latanoprost-loaded punctal plugs versus Xalatan© eye drops treatment in patients with open-angle early visual field defects, glaucoma, or ocular hypertension, with each treatment applied to a different eye.
	OTX-TP	Ocular Therapeutix Inc., Bedford, MA, USA	NCT02914509 (Phase 3, completed)	Travoprost	• Route: Punctum • This prospective, multicenter, randomized, parallel-arm, double-masked study aimed to assess the safety and efficacy of OTX-TP, a sustained-release drug product placed in the canaliculus of the eyelid, in individuals with open-angle glaucoma or ocular hypertension. Up to 550 subjects (1100 eyes) diagnosed with these conditions in both eyes received either OTX-TP or a placebo vehicle (PV) to evaluate OTX-TP’s safety and efficacy.
Contact lens	Dual drug-eluting contact lenses	University of Florida, Gainesville, FL, USA	NCT02852057 (Phase 1, completed)	Timolol maleate/ Dorzolamide hydrochloride	• Route: Ocular surface • The study aims to evaluate the safety and effectiveness of drug-eluting contact lenses for glaucoma therapy. These lenses will carry timolol maleate and dorzolamide hydrochloride, standard ophthalmic drugs, along with vitamin E, to facilitate extended drug release. Effectiveness will be measured by assessing the reduction in IOP following lens usage.
	latanoprost-eluting contact lens	Massachusetts Eye and Ear Infirmary, MA, USA	NCT04500574 (Phase 1, recruiting)	Latanoprost	• Route: Ocular surface • This study aims to evaluate the safety, comfort, and feasibility of reducing intraocular pressure using a novel Contact Lens Drug Delivery System containing latanoprost. Latanoprost, a well-established glaucoma medication, is typically administered as eye drops but presents challenges in adherence. This clinical trial investigates the safety, tolerability, and effectiveness of latanoprost delivery via a drug-eluting contact lens. The study comprises two phases: Phase A to evaluate safety and tolerability and Phase B to assess safety and effectiveness.
Subconjunctival injection	Eye-D VS-101	BioLight Life Sciences, Tel Aviv, Israel	NCT02129673 (Phase 1/2, completed)	Latanoprost	• Route: Subconjunctival insert • This Phase 1/2 multicenter study aims to evaluate the safety and ocular hypotensive efficacy of three dose levels of the VS101 Subconjunctival Latanoprost Insert over three months in subjects with open-angle glaucoma or ocular hypertension.
Intracameral implant	ENV515	Aerie Pharmaceuticals, Durham, NC, USA	NCT02371746 (Phase 3, completed)	Travoprost	• Route: Biodegradable intracameral implant • This multicenter, three-stage, open-label, prospective, active-comparator-controlled Phase 2a study investigates ENV515 (Travoprost) Intracameral Implant in patients diagnosed with bilateral ocular hypertension or early primary open-angle glaucoma.
	DURYSTA (BimSR)	Allergan, Dublin, Ireland	NCT02250651, NCT02247804, NCT04285580 (Phase 3, completed)	Bimatoprost	• Route: Biodegradable intracameral implant • These studies aims to assess the efficacy and safety of bimatoprost sustained-release in patients diagnosed with open-angle glaucoma or ocular hypertension. The trial comprises a 12-month treatment phase followed by an 8-month extended follow-up period.
	iDose	Glaukos, San Clemente, CA, USA	NCT02754596 (Phase 2, completed)	Travoprost	• Route: Noniodegradable intracameral implant • This randomized trial compares two elution doses of the Travoprost Intraocular implant to timolol ophthalmic solution in patients diagnosed with open-angle glaucoma. Study assessments will include measurements of IOP and medication use, along with safety parameters.
	OTX-TIC	Ocular Therapeutix Inc., Bedford, MA, USA	NCT05335122 (Phase 2, active)	Travoprost	• Route: Biodegradable intracameral implant • This is a prospective, multicenter, randomized, parallel-group, controlled study designed to assess the efficacy and safety of Travoprost intracameral implant in individuals diagnosed with OAG or OHT. Approximately 105 subjects will be enrolled across approximately 20 sites in the US. Participants will be randomly assigned to one of three treatment groups: OTX-TIC drug product (comprising two travoprost dose strengths) compared to a single injection of DurystaTM. Nonstudy eyes will receive treatment with a prostaglandin analogue if not contraindicated.

## Conclusion

A diverse array of ocular drug delivery systems has been developed for glaucoma treatment, featuring an expanding focus on long-acting implants, microspheres, and nanocarriers. These systems are increasingly favoured for their ability to offer sustained and controlled drug release, which is critical for improving patient compliance and therapeutic efficacy. The incorporation of neuroprotective agents, such as NO and H2S donors, represents a promising advancement in preventing further damage to RGCs. Most of these innovative systems have been rigorously tested for their physicochemical properties and evaluated through in vivo assays in animal models to assess ocular tolerance, drug pharmacokinetics, effectiveness, and sustainability of drug release over extended periods. Despite their advantages, many systems are associated with challenges such as invasiveness and potential postoperative irritation. However, the benefits of sustained drug release, which can span from days to months, offer significant improvements in patient adherence, particularly when tested in clinical trials. The exploration of additional sustained release components, such as biodegradable matrices or encapsulated drug reservoirs, continues to evolve, potentially enhancing the longevity and safety of these delivery systems. Recent clinical trials have demonstrated the feasibility and safety of these novel drug delivery platforms, confirming their potential to significantly benefit patients with glaucoma and ocular hypertension (OHT). As technology advances, the design of long-acting drug delivery systems must carefully consider several critical parameters, including the dimensions of the implants, desired release durations, rates of implant degradation, and comprehensive safety profiles, to fully optimize treatment outcomes and patient well-being in clinical settings.

## Data Availability

Data sharing is not applicable to this article, as no data set was generated or analysed during the current study.
